# Women’s knowledge about Intrauterine Device and user satisfaction in Brazil: a systematic review

**DOI:** 10.1590/1980-220X-REEUSP-2024-0262en

**Published:** 2025-05-26

**Authors:** Clarissa Oliveira Guimarães Lopes de Sousa, Síntia Nascimento dos Reis, Gisele Nepomuceno de Andrade, Bruna Nicole Soares dos Santos, Mariana Santos Felisbino-Mendes

**Affiliations:** 1 Universidade Federal de Minas Gerais, Escola de Enfermagem, Programa de Pós-Graduação em Enfermagem, Belo Horizonte, MG, Brazil.; 2 Universidade Federal de Minas Gerais, Escola de Enfermagem, Departamento de Enfermagem Materno-Infantil e Saúde Pública, Belo Horizonte, MG, Brazil.

**Keywords:** Knowledge, Patient Satisfaction, Intrauterine Devices, Systematic Review, Women’s Health

## Abstract

**Objective:**

To identify women’s knowledge about Intrauterine Device (IUD) and user satisfaction with the method in Brazil.

**Method:**

A systematic review, with searches carried out in the PubMed, Scopus, Web of Science and LILACS bibliographic databases via the Virtual Health Library Regional Portal in June 2023. The research followed the Preferred Reporting Items for Systematic reviews and Meta-Analyses guidelines, and the risk of bias was measured according to JBI criteria. Studies with no restriction on publication time, with a qualitative or quantitative methodological approach, assessing the satisfaction and knowledge about IUD of Brazilian women were selected.

**Results:**

A total of 1,215 articles were identified; of these, 12 met the final inclusion criteria, dated 1997–2023, seven on satisfaction with IUD use, and five related to knowledge about the method. No study addressed both outcomes simultaneously. Inadequate knowledge about IUD was observed, permeated by misconceptions. Women’s satisfaction with IUD was greater than 80% in most studies.

**Conclusion:**

The findings point to the stigmatization of IUD and gaps in knowledge about the method, in addition to showing a high rate of satisfaction among users of the method.

## INTRODUCTION

In Brazil, more than 80% of women use some contraceptive method (CM)^(1,2)^. However, the levels of unintended pregnancies in the country remain high. The Birth in Brazil survey, conducted with more than 20,000 women between 2011 and 2012, showed a prevalence of 55.4% of unintended pregnancies^(3)^. In a more recent study carried out in eight university hospitals, in the five regions of Brazil, which assessed 1,120 postpartum women, a proportion of unintended pregnancies of 67.4% was identified^(4)^. These pregnancies are associated with increased maternal and child morbidity and mortality^(5)^, and could be avoided with greater access to information and consistent use of the chosen CM.

The occurrence of an unintended pregnancy may occur due to contraceptive vulnerability favored by the high discontinuation rate, depending on the CM adopted^(6)^. Discontinuation is more frequent among users of short-acting contraceptive methods than among those using long-acting contraceptive methods (LARCs), 56.8% and 17.8%, respectively^(7)^. Among the LARCs, is the Intrauterine Device (IUD), a reversible method with a high degree of effectiveness^(8,9)^. Its effectiveness can be compared with that of definitive surgical methods, such as tubal ligation and vasectomy^(10)^, contributing positively to women’s health and public health by reducing the incidence of unintended pregnancies^(11)^.

In 2020, IUD was a method used by more than 161 million women worldwide^(6)^. Currently, countries such as China, Egypt and Tunisia have the highest rates of use of this method^(6)^. Between 1995 and 2020, the proportion of IUD users declined globally from 22% to 17%, with North Africa and West Asia seeing the largest decline^(6)^. On a smaller scale, Latin America also showed a decrease in the proportion of IUD use^(6)^, even though the use of this method in some countries on the continent has reached higher levels of access and use.

Brazil, for instance, saw an increase in the use of the method between 1990 and 2019 among women of reproductive age, from 1.8%^(2)^ to 4.4%^(7)^, with a further increase until 2030^(12)^. This increase may be the result of public policies aimed at reproductive planning. Among these, the following stand out: the Pact for Health, regulated by the Ministry of Health (MoH) Ordinance 399 of February 2006, which included financial investment to expand the range of contraceptives; the Stork Network, established within the scope of the Brazilian Health System (In Portuguese, *Sistema Único de Saúde* - SUS) by MoH Ordinance 1,459 of June 2011, which aimed to ensure the right to reproductive planning; and MoH Ordinance 3,265 of December 2017, which provides for the free provision of copper IUD for contraception after normal birth, cesarean section or immediate post-abortion in maternity hospitals in the country.

However, the use of IUDs in Brazil remains incipient, even with a decline in the use of the method globally^(6)^ and with the expected availability in SUS^(13)^. The reasons for the non-use of IUDs have been described and involve several organizational and individual factors. Among the organizational factors are the method insufficient supply, the recurrent application of criteria that are not characterized as method contraindications^(14)^, healthcare professionals’ inadequate knowledge about IUDs^(14,15)^ and the centralization of insertion in the hands of medical professionals^(14)^, even though the MoH resolution, through Technical Note 31/2023, provides for the possibility of IUDs being inserted by doctors and nurses, as long as they are qualified to do so^(13)^.

Among the individual factors for not using IUD, low knowledge about factors intrinsic to the method, such as its effectiveness, side effects, safety and mechanism of action, stands out^(16)^. It is also worth considering the misconceptions that create an atmosphere of distrust and hesitation regarding the method. A study conducted in Ethiopia with women who had an IUD inserted after giving birth found that women with greater knowledge about IUD were almost four times more likely to use the method, when compared to those with less knowledge^(17)^.

Furthermore, adequate knowledge about the method can positively impact satisfaction with use, as it is assumed that users will be able to choose the method that best suits their needs and expectations^(18)^. Furthermore, satisfaction with the adopted method is related to greater continuity of use^(19)^ and, consequently, to a reduction in contraceptive vulnerability^(6)^. Satisfaction with IUD use is high worldwide. A study conducted with women from 11 European countries found that 78% and 76% were very satisfied with hormonal and copper IUDs, respectively^(20)^. It is worth noting, however, that despite the presence of myths and inadequate knowledge among the Egyptian population^(21)^ regarding IUD, users reported high satisfaction (77.8%)^(22)^, and the country remains among those whose women use the method the most^(6)^.

Given these data, it is important to summarize the evidence related to knowledge about IUD and satisfaction with the use of the method in Brazil to identify possible characteristics of the Brazilian population that may justify the lower use of the method in the country. Moreover, summarizing this evidence could contribute to a better understanding of these fundamental aspects in a context of underuse of the method, less than 5%, in Brazil. The proposed summary could also contribute to developing actions to improve access and correct use of the method. Thus, this systematic review aims to identify women’s knowledge about IUD and users’ satisfaction with the method in Brazil.

## METHOD

### Study Design

This is a systematic review developed to answer the question “What is women’s knowledge about IUD and how satisfied are the method users in Brazil?”. The stages for reporting the systematic review followed the Preferred Reporting Items for Systematic reviews and Meta-Analyses (PRISMA) guidelines^(23)^: 1) topic identification and hypothesis or research question selection; 2) establishment of criteria for study inclusion and exclusion; 3) definition of information to be extracted from selected studies; 4) assessment of studies included in the review; 5) interpretation of results; and 6) presentation of review/synthesis of knowledge. This study also followed the Sex and Gender Equity in Research (SAGER) guidelines^(24)^, which provide for the cautious use of sex and gender in scientific publications and standardization of these definitions. In this study, we included as search categories cisgender women (assigned female at birth and who identify as women), transgender men (assigned female at birth but who identify as men), and non-binary people with a uterus (assigned female at birth but who identify as neither men nor women), regardless of age.

### Eligibility Criteria

Primary studies in English, Spanish, Portuguese, and French that were available in full, without restriction on publication date, with a qualitative and/or quantitative methodological approach that assessed satisfaction or knowledge about IUD of people with a uterus (cisgender women: assigned female at birth and who identify as women; transgender men: assigned female at birth but who identify as men; and non-binary people with a uterus: assigned female at birth but who identify as neither men nor women), regardless of age, and who lived in Brazil, were included. In satisfaction studies, current or past use of IUD as a CM was mandatory. In contrast, articles that investigated knowledge about IUD in both the method users and non-users were considered eligible.

Studies that considered “knowledge” as purely knowing about the existence of IUD as a CM or that studied healthcare professionals’ knowledge about the method were excluded. Systematic reviews, case reports, reviews, editorials and ministerial documents were also excluded.

### Protocol Registration

The protocol containing the stages of the review and synthesis of findings was registered and published in the International Prospective Register of Systematic Reviews (PROSPERO) (http://www.crd.york.ac.uk/PROSPERO), under CRD42024611232.

### Search Strategy

The mnemonic PECO was used (P: population/patients; E: exposure; C: comparison/control; O: outcome)^(25)^. In this study, population consisted of people with a uterus; exposure was the use or assessment of IUD; and outcomes were satisfaction and knowledge regarding the method. The search strategy was developed using descriptors and their alternative terms taken from the classification systems of terms for indexing: Health Science Descriptors (DeCS), using four languages (Portuguese, English, Spanish and French); and the Medical Subject Headings (MeSH), for the search in English.

The databases used in the bibliographic search were PubMed, Scopus, Web of Science (WoS) and LILACS via the Virtual Health Library (VHL). The search in the Scopus and WoS databases was carried out through the Coordination for the Improvement of Higher Education Personnel (In Portuguese, *Coordenação de Aperfeiçoamento de Pessoal de Nível Superior* - CAPES) journal portal consortium. The search was carried out in June 2023, with no restrictions on publication date or other filters.

The descriptors were combined using Boolean operators (OR and AND), respecting the search characteristics of each database. The free search in WoS allows a maximum of 50 terms. Therefore, the strategy was reduced by using “*” for terms with variations. Furthermore, an additional search was performed in the reference lists of studies included in the review. The search strategy used in VHL included terms in Portuguese, English, Spanish and French. In the other databases, only terms in English were used. The search strategy is described below: (*Mulher* OR *Mulheres* OR *Meninas* OR *Adolescente* OR *Adolescentes* OR *Jovens* OR *Jovem* OR Women OR Girls OR Girl OR Woman OR Adolescent OR Adolescents OR Teens OR Teen OR Teenagers OR Teenager OR *Mujeres* OR *Femmes* OR “*Pessoas Transgênero*” OR “*Homem Transexual*” OR “*Homens Trans*” OR “Transgender Persons” OR “Transgender Man” OR “Trans Man” OR “Transgender Men” OR “*Personas Transgénero*” OR “*Personnes transgenres*” OR “*Pessoas Não-binárias*” OR “Non-binary People” OR “Non-binary Person” OR “*Personas No Binarias*” OR “*Personnes Non Binaires*”) AND (“*Dispositivos Intrauterinos*” OR “*Anticoncepcionais Intrauterinos*” OR DIU OR “*Sistema Intrauterino*” OR SIU OR “DIU Hormonal” OR “DIU de Cobre” OR “Intrauterine System” OR IUS OR “Intrauterine Devices” OR “Device, Intrauterine” OR “Devices, Intrauterine” OR “Intrauterine Device” OR “Contraceptive IUDs” OR “Contraceptive IUD” OR “IUD, Contraceptive” OR “IUDs, Contraceptive” OR “Contraceptive Devices, Intrauterine” OR “Contraceptive Device, Intrauterine” OR “Device, Intrauterine Contraceptive” OR “Devices, Intrauterine Contraceptive” OR “Intrauterine Contraceptive Device” OR “Intrauterine Contraceptive Devices” OR “Unmedicated IUDs” OR “Unmedicated IUD” OR “IUD, Unmedicated” OR “Intrauterine Devices, Medicated” OR “Device, Medicated Intrauterine” OR “Devices, Medicated Intrauterine” OR “Intrauterine Device, Medicated” OR “Medicated Intrauterine Device” OR “Intrauterine Devices, Hormone-Releasing” OR “Device, Hormone-Releasing Intrauterine” OR “Devices, Hormone-Releasing Intrauterine” OR “Hormone-Releasing Intrauterine Device” OR “Hormone-Releasing Intrauterine Devices” OR “Intrauterine Device, Hormone-Releasing” OR “Intrauterine Devices, Hormone Releasing” OR “IUD, Hormone Releasing” OR “Hormone Releasing IUD” OR “Medicated Intrauterine Devices” OR “Hormone-Releasing IUDs” OR “Hormone-Releasing IUD” OR “IUD, Hormone-Releasing” OR “IUDs, Hormone-Releasing” OR “Intrauterine Devices, Copper” OR “Copper Intrauterine Device” OR “Device, Copper Intrauterine” OR “Devices, Copper Intrauterine” OR “Intrauterine Device, Copper” OR “Copper-Releasing IUDs” OR “Copper-Releasing IUD” OR “IUD, Copper-Releasing” OR “IUDs, Copper-Releasing” OR “IUD, Copper Releasing” OR “Copper Releasing IUD” OR “Copper Intrauterine Devices” OR “*Dispositifs Intra-utérins*” OR IUD OR “*Système Intra-utérin*” OR “*Métodos Contraceptivos*” OR “MC” OR “Contraceptive Device” OR Contraception OR “Contraceptive Methods” OR “Contraceptive Method” OR “*Métodos Anticonceptivos*” OR “*Méthodes Contraceptives*”) AND (“*Satisfação do Paciente*” OR *Satisfação* OR “Patient Satisfaction” OR Satisfaction OR “*Satisfacción del Paciente*” OR “*Satisfaction des Patient*s” OR *Conhecimento* OR *Conhecimentos* OR Knowledge OR *Conocimiento* OR *Savoir*) AND (*Brasil* OR Brazil OR Brésil).

### Study Selection

The search was conducted in June 2023 and retrieved 1,215 articles. The studies found were exported to the Rayyan platform, a free online platform used to manage articles in the construction of systematic reviews, integrative reviews and other types of evidence syntheses to exclude duplicates and select studies in three stages. The first stage of the process was conducted by two independent reviewers (COGLS and SNR), who read the titles and abstracts of each article to select the studies according to the previously established criteria. Disagreements between these reviewers (n = 26) were broken by a third reviewer (MSFM). Finally, the selected articles were read in full for final inclusion.

After the complete reading, the titles of selected studies were searched on the Retraction Watch website (https://retractionwatch.com/), which identifies retracted articles that are available in databases such as Scopus, National Library of Medicine, among others. Moreover, research was carried out on the journals in which the studies selected in Beall’s List were published (https://beallslist.net), which identifies journals and publishers with predatory practices.

### Data Extraction

For data extraction, a spreadsheet was created in Excel/Microsoft software, and the process was conducted again by two reviewers (COGLS and SNR), independently. The following information was extracted from the selected studies: 1) characteristics related to the study: title, authors, year of publication, language, location, journal of publication and time of collection; 2) methodological attributes: objectives, study design, sample size, data collection method and population; 3) population characteristics: age range and whether or not they use the method; and 4) outcomes of interest: women’s knowledge about IUD and users’ satisfaction with the method.

The findings of the two reviewers were compared in order to identify possible discrepancies and assess the consistency of information collected. Disagreements between the results were resolved by consensus.

### Data Analysis

The assessment of the methodological quality of selected studies was carried out using JBI criteria to verify the risk of bias and how it was addressed by the authors in the study^(26)^. Quantitative studies were assessed using a questionnaire for prevalence/incidence research, which contains nine questions; and qualitative studies were assessed using a checklist for qualitative studies, with ten questions. In both instruments, for each question, the answer could be “yes”, “no”, “unclear” or “not applicable”^(26)^. The more “yes” votes a study receives, the higher the score, which indicates a lower risk of bias.

Prevalence studies that received “yes” to seven or more questions were classified as having high methodological quality; those that received “yes” to five to six of the questions were classified as having average methodological quality; and studies that received “yes” to four or fewer were classified as having low methodological quality. In contrast, qualitative studies that received “yes” to eight or more questions were classified as having high methodological quality; those that received “yes” to five to seven of the questions were classified as having average methodological quality; and studies that received “yes” to four or fewer were classified as having low methodological quality. Assessment was performed by two reviewers (COGLS and SNR) independently. The results were compared, and disagreements were resolved by consensus.

Concerning extracted data analysis, a qualitative synthesis was performed. The methodological attributes of studies, the measured outcome, and main results raised were considered to create larger categories (knowledge and satisfaction) and smaller categories (instruments to measure knowledge and reasons related to satisfaction or dissatisfaction), according to the recurrence of the findings. This categorization resulted from the thematic analysis^(27)^ of articles included in the review.

## RESULTS

### Study Selection

**Figure 1:**
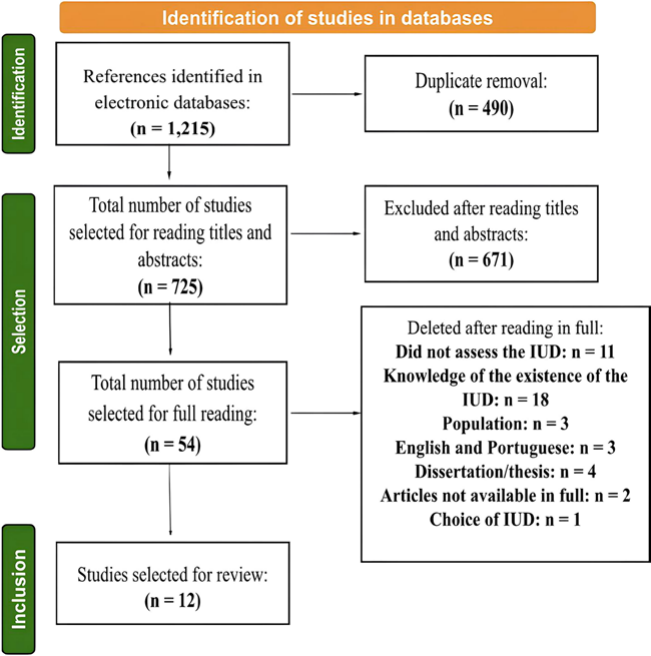
Flowchart of study selection for review – Brazil, 2024.

Of the 1,215 studies found, 490 were excluded due to duplication, leaving 725 articles (Figure 1). After reading the titles and abstracts, 671 studies were excluded because they did not meet the inclusion criteria, leaving 54 articles. After the full reading, 42 studies were excluded because they also did not meet the inclusion criteria, namely: 11 did not discuss IUD, but rather other CM; 18 investigated whether the study population only knew about the existence of IUD; three did not meet the inclusion criteria established for the population of this study (one measuring outcomes in healthcare professionals, one with a sample of non-Brazilian people and one with a population of cisgender men); three were duplicates, written in Portuguese and English; four were dissertations (three) or theses (one), whose articles had already been published; two were not found in full for free; and one article studied only the reasons for choosing IUD as a CM and did not address the outcomes of interest in this review. Thus, 12 articles were included in this review. None of the selected articles were retracted, and none of the publishing journals was considered predatory.

Of the scientific studies found, five discussed aspects related to knowledge about the method (Tables 1 and 2), while the other seven studied user satisfaction with IUD (Table 3). No article assessed both outcomes simultaneously, nor did it have trans men or non-binary people as the study population. Therefore, the present study will only discuss results found in populations of cisgender women and will therefore use feminine pronouns to refer to the findings.

**Table 1:** Characteristics of studies on women’s knowledge about Intrauterine Device – Brazil, 2024.

First author/year and citation number	Population/sample	IUD use	Methodological approach and study design	Place	JBI**
Camiá/1997(^28^)	100 postpartum women	No	Qualitative with discourse analysis	Campo Grande, MS	6/10
Espejo/2003(^31^)	472 women between 30 and 49 years old	No	Quantitative cross-sectional	Campinas, SP	9/9
Silva-Filho/2016(^30^)	380 women between 20 and 30 years old	s/e*	Quantitative cross-sectional	Brasil	4/9
Diniz/2020(^29^)	22 women between 14 and 24 years old	No	Qualitative with thematic analysis	São Luís, MA, Balsas, MA, e Santana do Ipanema, AL	7/10
Borges/2020(^16^)	1.858 women aged 18–49	s/e*	Cross-sectional quantitative	São Paulo, SP, Aracaju, SE e Cuiabá, MT	8/9

Note: *No specification;

**Verification of risk of bias using JBI criteria; IUD – Intrauterine Device; MS – Mato Grosso do Sul; SP – São Paulo; MA – Maranhão; AL – Alagoas; SE – Sergipe; MT – Mato Grosso.

**Table 2:** Knowledge about Intrauterine Device among Brazilian women who use or do not use the contraceptive method – Brazil, 2024.

First author/year and citation number	Objective	Instrument	Question(s)	Main results
Camiá/1997(^28^)	Assess the level of information of postpartum women regarding contraceptive methods.	Structured questionnaire with ten open-ended and closed-ended questions. No specific question about IUD, and it could be mentioned freely.	Have you heard how to prevent pregnancy? (yes/no); What have you heard about preventing pregnancy? (open); What do you know? (open to talk about the methods mentioned).	Correct: 19.4%* Partially correct: 41.9%* Incorrect: 38.7%*
Espejo/2003(^31^)	Assess knowledge about CM and their possible association with socioeconomic and demographic characteristics.	Structured questionnaire with 18 questions, six of which were statements about contraceptive methods and, of these, only one about IUD.	When a woman uses an IUD and becomes pregnant, the baby is born with IUD attached to it (Closed - agree, disagree, or more or less).	They responded that, in case of failure, the baby will be born with IUD stuck to it (no percentage described).
Silva-Filho/ 2016(30)	Create knowledge data of Latin American women about IUD.	Online survey with eight statements about IUD with “yes” or “no” response options.	1 – Have heard negative stories about IUDs on TV, in magazines or newspapers.	About 30% reported hearing negative stories about IUD from friends and family.
			2 – Have heard negative stories about IUDs on the internet.	About 10% reported hearing negative stories about IUD from healthcare professionals.
			3 – Have heard negative stories about IUD from friends and family.	Around 25% believe that IUD cannot be used by nulliparous women.
			4 – Have heard negative stories about IUDs from healthcare professionals.	About 15% of women agreed that IUD is more effective than other contraceptive methods.
			5 – It can only be used by women who have already had children.	About 40% agreed that it is a more expensive method than most other contraceptive methods.
			6 – Can only be used by older women (40+).	The other results had a p-value > 0.05.
			7 – It is more effective than other contraceptive methods.	
			8 – It is more expensive than most other CM contraceptives.	
Diniz/2020(29)	Describe the perception and needs of young women seeking sexual and reproductive healthcare services.	Semi-structured interview that followed a topic guide**.	Questions raised about the Zika virus and its sexual transmission, as well as contraception, abortion, pregnancy and sexual and reproductive health needs.	Women reported fear of inclusion because they had heard stories in their communities.
				Cancer, miscarriage, pelvic inflammatory disease, infertility, and pain have been associated with IUD use by women.
				Furthermore, women reported fear of inserting a foreign object into their bodies.
Borges/2020(16)	Analyze the level of knowledge about IUD among women of reproductive age.	Structured questionnaire with six statements about IUD with response options “agree”, “disagree”, “do not know”.	1 – The man feels IUD during sexual intercourse.	1 – 53.7% did not know whether the man feels IUD during sexual intercourse and 4.9% agreed.
			2 – IUD is inserted through surgery.	2 – 24.3% believed that IUD is inserted through surgery and 26.1% did not know how to answer.
			3 – IUD is abortive.	3 – 13.7% agreed that IUD is abortive and 32.2% did not know how to answer.
			4 – After IUD is removed, the woman has difficulty becoming pregnant.	4 – 13.3% agreed that women will have difficulty becoming pregnant after removing IUD and 33.8% did not know how to answer.
			5 – IUD increases the risk of uterine cancer.	5 – 21% believed that IUD increases the risk of uterine cancer and 41.4% did not know how to answer.
			6 – IUD causes many unpleasant side effects.	6 – 31.3% agreed that IUD causes many unpleasant side effects and 39.8% did not know how to answer.
				Note: 7.2% of women answered all six items correctly and 15.3% answered all six items incorrectly. The median number of correct answers was 3.

Note: IUD – Intrauterine Device; n^º^ – number;

*Classification extracted from the original article;

**The questions asked according to each topic were not described in the original article.

**Table 3:** Satisfaction with the use of Intrauterine Device by Brazilian women using the method – Brazil, 2024.

First author/year and citation number	Population/sample	Methodological approach and study design	Place	Instrument	Main result	JBI**
Albuquerque/2021(^32^)	158 postpartum women	Quantitative, cohort, prospective	Fortaleza, CE	Questionnaire with Likert scales	6 wk: 92.4% satisfied 6 m: 86.9% satisfied	6/9
Scavuzzi/2016(^33^)	157 nulliparous women and women with previous births	Quantitative, cross-sectional	Recife, PE	Structured questionnaire with 0–10 satisfaction scale	Nulliparous women: 77.8% completely satisfied With previous birth: 76.7% completely satisfied	6/9
Laporte/2022(^38^)	517 women using hormonal IUD	Quantitative, cross-sectional	Campinas, SP	Closed-ended questionnaire	91% of women satisfied with IUD 97% would recommend it to other women	4/9
Oliveira/2023(^34^)	100 women between 15 and 24 years old	Quantitative, cohort, prospective	Belo Horizonte, MG	Structured questionnaire	12 months: 93.9% satisfied 24 months: 100% satisfied 36 months: 96.8% satisfied	5/9
Carvalho/2017(^35^)	251 women using hormonal IUDs between 18 and 45 years old	Quantitative, cohort, prospective	Campinas, SP	Structured questionnaire	12 months: 93.1% very satisfied or satisfied*	5/9
Giovanelli/2021(^36^)	294 adolescents < 19 years old	Quantitative, cohort, prospective	São Bernardo do Campo, SP	Structured questionnaire	12 m: 85.7% satisfied*	5/9
Borges/2017(^37^)	668 women aged 18–49	Quantitative, cross-sectional	São Paulo, SP	Structured questionnaire	IUD users: 94.7% satisfied	9/9

Note: m – months; wk – weeks; CM – contraceptive methods;

*Although the study was defined by the authors as a prospective cohort, the outcome of interest in this review was measured at only one-time point;

**Verification of risk of bias using JBI criteria.

### Knowledge

The five studies on women’s knowledge about IUDs were dated from 1997 to 2020, and only two were published in the last five years (Table 1). Regarding the methodological approach, three were cross-sectional quantitative studies and two were qualitative, one with thematic analysis and the other with discourse analysis. The studies investigated knowledge in the method non-users (n = 3) and in women who used or did not use IUDs (n = 2).

The sample size of these studies rAnged from 22 to 1,858 women. The studies assessed women between 14 and 49 years of age, but each with a different age range. Only one study considered the sample collected in Brazil, regardless of the region. The others were studies with local or regional samples, such as interstate ( n = 2) or within a single municipality (n = 2). Of the studies conducted in municipalities, one was from the state of São Paulo, and one from Mato Grosso do Sul (Table 1).

In studies that assessed knowledge about IUD, misinformation about the method was evident, such as beliefs that IUD: is abortive^(16,28,29)^; causes cancer^(28,29)^, pelvic inflammatory disease^(29,30)^ and ectopic pregnancy^(30)^; makes it difficult for a woman to become pregnant after removal or causes infertility^(16)^; it is only recommended for women who have already had child(ren)^(30)^; and that, in case of failure, the baby will be born with IUD stuck to it^(31)^ (Table 2).

Women reported hearing negative stories about IUD from friends, family and even healthcare professionals^(29,30)^. Furthermore, fear of insertion, of introducing a foreign object into the body and of pain at the time of insertion or during use was reported by women as reasons for fear of adopting the method^(29)^. Gaps in knowledge about where IUD is inserted^(28)^ and what the method is, with women believing it to be a ring or disc, for instance^(28)^, were found.

In a qualitative study^(28)^, categories were created by the authors so that the answers could be considered correct, partially correct and incorrect. In this study, women were considered correct when they said that IUD is a device placed in the uterus by the doctor and that it should be periodically checked. They were considered partially correct when reported that the IUD was inserted inside the woman, in the cervix or vagina, that it was something similar to a fishing line with two ends and that it was abortive. Finally, they considered incorrect when they considered that IUD was similar to a skin, that it prevented pregnancy and diseases; that it was a ring covered in ointment to be inserted into the uterus; and that it was a disk placed at the entrance of the uterus that acts as a lid. Considering these categories, 19.4% presented correct knowledge about IUD, 41.9% partially correct, and 38.7% incorrect (Table 2).

A study that investigated health unit users’ knowledge about IUDs^(16)^ asked six questions, to which women could respond with “agree”, “disagree” or “do not know”, namely: 1) IUD causes abortion; 2) after IUD is removed, women have difficulty becoming pregnant; 3) IUD is inserted through surgery; 4) men can feel IUD during sexual intercourse; 5) IUD increases the risk of uterine cancer; 6) IUD causes many unpleasant side effects. Of the 1,858 women who made up the sample, only 7.2% answered all six items correctly and 15.3% answered all six items incorrectly, and the median number of correct answers was three (Table 2).

### Satisfaction

Seven studies investigated user satisfaction with the method and were published between 2016 and 2022, four of them in the last five years (Table 3). As for the methodological approach, all studies are quantitative, with a predominance of prospective studies (n = 4), followed by cross-sectional studies (n = 3). The sample size of studies varied between 100 and 668 women. The studies analyzed women aged between 15 and 49 years, each with different age groups. All used local samples at the municipal level. Of these, four studies were conducted in the state of São Paulo, one in Minas Gerais, one in Ceará and one in Pernambuco.

High percentages of satisfaction with IUD use were found in studies with the Brazilian population of the method users, namely: 92.4% and 86.9% were satisfied after six weeks and six months of insertion, respectively^(32)^; 77.8% of nulliparous women and 76.7% of women with a previous birth were completely satisfied^(33)^; 93.9%, 100% and 96.8% satisfied with 12, 24 and 36 months of use, respectively^(34)^; 93.1% satisfied 12 months after insertion^(35)^; 85.7% satisfied 12 months after giving birth^(36)^; and 94.7%^(37)^ and 91%^(38)^ satisfied, regardless of the time of use.

Among the seven articles that studied user satisfaction, four investigated factors related to dissatisfaction with IUD use; two studies discussed factors related to satisfaction with the method; and one study discussed the reasons for satisfaction and dissatisfaction. The results are summarized in Table 4.

**Table 4:** Factors related to (dis)satisfaction with the use of Intrauterine Device among Brazilian women – Brazil, 2024.

Dissatisfaction	Citation number
Increased or prolonged menstrual bleeding	(32,35,36)
Dysmenorrhea	(32,36)
Breakthrough bleeding	(35)
Other side effects (such as acne, weight gain, and migraines)	(35)
**Satisfaction**	**Citation number**
Amenorrhea or decreased menstrual flow	(35,38)
Confidence in the method	(33)
Reduction in dysmenorrhea	(38)
Consultation with a healthcare professional before using the method	(37)

## DISCUSSION

The findings of this study point to the stigmatization of IUD and gaps in knowledge about it, factors that may impact women’s desire to use the method in the country. It is worth noting the lack of standardization of questions or aspects to be measured to assess knowledge about the method, which, to some extent, compromises the comparison of results of existing studies. However, these findings in Brazilian studies corroborate those of a systematic review carried out with studies from other countries, in which women also associated IUD with infertility, ectopic pregnancy and cancer, in addition to the belief that the method cannot be used by nulliparous women, for instance, especially in low- and middle-income countries in Asia and Africa^(39)^.

In this summary, the reasons for not using the method were the fear of introducing a foreign object into the body and the fear of the insertion procedure. These findings corroborate a study carried out in Korea that investigated women’s perceptions about IUD^(40)^. Additionally, Brazilian women reported fear of pain during insertion, which led them to not use it. However, a study conducted in São Paulo with women who underwent IUD insertion showed that less than 19% classified pain as intense, and the majority classified pain as moderate (44%) and mild (37.3%)^(41)^. On the other hand, pain is an individual experience and may be present during IUD insertion, and professionals should inform women about strategies to mitigate it, including offering analgesia^(42)^.

The lack of this knowledge and practice may also contribute to lower use of the method. Additionally, the findings also reinforce that the lack of knowledge and misconceptions about the method and its insertion may encourage exaggerated fears, causing harm to women themselves because they do not use the method for these reasons. Thus, the results found on inadequate knowledge about IUD by Brazilian women indicate that access to reproductive planning services, especially access to adequate information about this method, appears to be below the recommended level^(43)^.

Despite the low and misleading knowledge about the method, it is worth noting that women are interested in receiving more information. The Thinking About Needs in Contraception study, an online quantitative survey on healthcare professionals’ and women’s opinions regarding aspects of contraceptive counseling and contraceptive use, found that 66% of women were interested in receiving more information about all methods and that 69% would consider using LARCs if they received more comprehensive information^(44)^. Thus, it is reiterated that, if women received adequate information, the concerns and fears reported in the studies could be alleviated.

Even with the scarce assessment of the source of misinformation about IUD in the studies, it is not unrealistic to assume that women may have sought answers to their questions on the internet. In this context, it is necessary to consider the unreliability of these websites, which, even when specialized in contraception, disseminate inconsistent and misleading information about the method, which may contribute to non-use of the method^(45)^. On the other hand, when information is provided by trained healthcare professionals, interest in using it increases. A study conducted in São Paulo found that more than 98% of women who had IUD inserted by midwives and obstetric nurses reported that they received guidance on reproductive planning before insertion and that, of these, practically all (98.6%) considered that it was sufficient for them to feel safe using IUD^(41)^, even though studies in this review showed that women recognize medical professionals as the one responsible for insertion.

Trained nurses have support to insert IUD^(13)^, and, within the scope of nursing, they have exclusive competence to work in family and reproductive planning^(46)^. Additionally, a study carried out with data from the Health Information System for Primary Care in 2021 included nurses who carry out the majority (approximately 76%) of individual consultations related to IUD, while medical professionals carried out approximately 24% of consultations for guidance on the method^(47)^. A study conducted in Ethiopia investigated factors associated with postpartum IUD use and found that women who received professional guidance after giving birth were three times more likely to use IUD than those who did not receive such guidance^(17)^.

Thus, it is clear that there is a clear need for healthcare professionals to provide women with accurate information and scientific basis. However, the literature discusses the existence of a knowledge gap presented by some of these professionals regarding IUD. A study carried out in Cape Town, South Africa, indicated that approximately 14% of professionals believed that IUD can migrate in the body, 11.1% that it increases the chance of miscarriage, 18.1% that it is associated with ectopic pregnancy, and 22.2% that it is associated with^(15)^.

Having access to scientifically based knowledge about CM, especially from trained professionals, is essential for women to be able to make decisions about their sexual and reproductive health, allowing them to choose the method that best suits their needs and life circumstances^(18)^. These educational actions, collective and individual counseling, configure what is recommended in the context of reproductive planning^(43)^. However, a study carried out with a representative sample of the Brazilian population found that 95.4% of women in 2019 had not participated in reproductive planning groups^(1)^. The North American CHOICE study showed that increased knowledge and the removal of financial barriers led to an increase in the use and rate of continuation and satisfaction with LARCs^(48)^, reinforcing the need for guidance.

The studies included in this review also showed a high satisfaction rate among IUD users. However, there was a lack of standardized parameters to assess the level of user satisfaction. Different instruments were used, such as a Likert scale, a 0–10 scale, and various categorizations (satisfied or dissatisfied; very satisfied, satisfied, dissatisfied, or very dissatisfied; and completely satisfied, partially satisfied, or dissatisfied), similar to what was observed for assessing knowledge about the method. This fact compromises, to some extent, the comparability of their findings among studies.

Among the side effects, changes in bleeding patterns were the most cited as a determinant of satisfaction with IUD use. Increased bleeding, in frequency or intensity, was a reason for dissatisfaction, while decreased bleeding was a positive factor for satisfaction. Hence, once again, prior knowledge could help women identify and manage these effects. However, such changes in bleeding patterns were not enough to reduce the satisfaction rate of Brazilian women with IUD use, as evidenced by surveys conducted in Brazil, which indicated, for the most part, over 80% satisfaction. These rates are even higher than those found in Europe, with 76–78% very satisfied, and in Egypt, 77.8% satisfied^(22)^.

It can be seen, therefore, that IUD is a LARC-type method widely used in high-income countries, but little used in Brazil. Brazilian women have little knowledge about the method and are surrounded by misconceptions, a reality that is also perceived worldwide, which could partly justify the underuse of the method in Brazil compared to the global context. On the other hand, when they do use it, the level of satisfaction is very high, even higher than in other countries. Based on the findings of this study, it can be inferred that there are problems with access, which includes access to information that may be insufficient, in addition to the availability of the method on the SUS menu.

Considering women’s interest in receiving more comprehensive information about the method^(44)^ and the impacts of this guidance on the likelihood of use^(17)^, healthcare professionals in Brazil can play a key role in ensuring access to adequate knowledge. In this regard, training of professionals also seems to be necessary, and the participation of nurses and nurses-midwives has been fundamental in ensuring the right and access to this and other methods^(41,47)^. Thus, it is expected that, after consultation with a trained professional, women’s level of knowledge will increase, also increasing the chances of using IUD. However, no study assessing Brazilian women’s knowledge about the method before and after consultation with a healthcare professional or educational intervention was found in the literature.

As a limitation of this study, evidence should be generalized with caution, due to the different designs and methods for measuring knowledge about the method and satisfaction with it among users. Furthermore, national production on the subject is concentrated in the city of São Paulo, with five (41.67%) of the 12 studies included being conducted there. This city belongs to the most developed state in the country and whose population could have considerable differences compared to that of the other states in the country, especially when considering inequalities in access to contraception previously described^(1,2)^. This results in different choices and uses of available CM by the population.

Furthermore, no studies have investigated outcomes in transgender men or non-binary people. Access for this population is even more restricted than for cisgender women, who have also been limited. A study indicates that marginalized groups have greater difficulty accessing sexual and reproductive healthcare services^(49)^. Therefore, it is not unrealistic to infer that this group has less access to information and, therefore, even lower knowledge than that found in the studies included in this review, which may result in a lower chance of use by trans men and non-binary people. Therefore, studies that assess knowledge in this population are necessary, in addition to strategies that are inclusive to ensure use.

Additionally, systematic reviews depend on the quality of the studies included. In the analysis of the methodological quality of studies, eight studies were classified as having average quality, mainly due to the lack of discussion of response rates (authors should clearly discuss the response rate and any reasons for non-response and compare people in the study with those not in the study), in addition to the use of different measures for knowledge and different scales for satisfaction. Therefore, there is a need for future studies to be strengthened methodologically and to allow greater comparability between them.

On the other hand, this study advances by identifying gaps in literature on assessment of women’s knowledge regarding IUD. Historically, studies have focused on myths about IUD and measured aspects related to women’s beliefs, without standardizing the findings. Thirty years ago, lack of knowledge about IUD included the physical appearance of the method, with women believing it to be a ring or disc, but these concepts have no longer been addressed and may be different today. Research from about ten years ago pointed to the belief that IUD is indicated only for women who have had children and that it can cause ectopic pregnancy or pelvic inflammatory disease.

The belief that IUD causes abortion and cancer was described in studies conducted in both 1997 and 2020, which indicates that this conception has been maintained over time. On the other hand, information on knowledge of side effects, duration, efficacy, and mechanism of action was not included, regardless of the time frame. Updated studies on the conceptions, knowledge, and satisfaction of people with uteruses who use IUD are necessary given: these identified gaps; older scientific evidence and the persistent lack of information about the method; the consequences of (not) using contraceptives, even when a person does not want to become pregnant; and the low prevalence of use of the method in Brazil.

Furthermore, by systematizing evidence on Brazilian women’s knowledge and satisfaction with the method, the study advances and may provide support for the development of actions to improve access to the method, which appears to involve obstacles beyond deficient knowledge. This reality should be considered, especially in Brazil, a country with a continental territory, historical social inequalities and great cultural and religious diversity, factors that may be related to the underuse of IUD in the country.

## CONCLUSION

This review allowed the synthesis of scientific evidence on Brazilian women’s knowledge about IUD and the satisfaction of users of this method. The findings indicate gaps in Brazilian women’s knowledge about IUD, highlighting misconceptions and myths that permeate it. The study points to the stigmatization of the method and the dissemination of erroneous beliefs, such as associations with infertility and cancer, including by professionals. These findings highlight the need to strengthen and improve guidance by healthcare professionals, including their training and aiming at the adequacy of knowledge based on promotion of access to accurate and scientifically based information. This guidance should be carried out in the context of the recommended reproductive planning actions that also need to be strengthened.

Despite this gap, women who used the method reported high satisfaction rates, despite prior concerns and misinformation. Although there are variations in the satisfaction assessment instruments, the results suggest that, once the initial barriers are overcome, IUD is widely accepted by Brazilian women. Ultimately, ensuring access to accurate information is essential to ensure women can make free and informed decisions about their reproductive health, promoting greater satisfaction and continued use.

## Data Availability

The entire dataset supporting the results of this study was published in the article itself.
