# Supplementary material for: Women’s knowledge about Intrauterine Device and user satisfaction in Brazil: a systematic review
Source: Rev Esc Enferm USP. 2025 May 26;59:e20240262. doi: 10.1590/1980-220X-REEUSP-2024-0262en (PMC12118360; doi:10.1590/1980-220X-REEUSP-2024-0262en)
Supplement: Translation

# Translation: Conhecimento de mulheres sobre o Dispositivo Intrauterino e a satisfação das usuárias no Brasil: revisão sistemática

**DOI:** 10.1590/1980-220X-REEUSP-2024-0262pt

**Authors:** Clarissa Oliveira Guimarães Lopes de Sousa, Síntia Nascimento dos Reis, Gisele Nepomuceno de Andrade, Bruna Nicole Soares dos Santos, Mariana Santos Felisbino-Mendes

**Affiliations:** 1 Universidade Federal de Minas Gerais, Escola de Enfermagem, Programa de Pós-Graduação em Enfermagem, Belo Horizonte, MG, Brasil.; 2 Universidade Federal de Minas Gerais, Escola de Enfermagem, Departamento de Enfermagem Materno-Infantil e Saúde Pública, Belo Horizonte, MG, Brasil.

**Keywords:** Conhecimento, Satisfação do Paciente, Dispositivo Intrauterino, Revisão Sistemática, Saúde da Mulher

## Abstract

**Objetivo:**

Identificar o conhecimento de mulheres sobre o Dispositivo Intrauterino (DIU) e a satisfação das usuárias com o método no Brasil.

**Método:**

Revisão sistemática, com busca realizada nas bases de dados bibliográficas PubMed, Scopus, *Web of Science* e LILACS via Portal Regional da Biblioteca Virtual em Saúde em junho/2023. A pesquisa seguiu diretrizes do *Preferred Reporting Items for Systematic reviews and Meta-Analyses*, e o risco de viés foi mensurado de acordo com critérios do JBI. Selecionados estudos sem restrição de tempo de publicação, com abordagem metodológica qualitativa ou quantitativa, avaliando a satisfação e o conhecimento sobre o DIU das brasileiras.

**Resultados:**

Identificados 1.215 artigos; desses, 12 atenderam aos critérios de inclusão final, datados de 1997-2023, sete sobre a satisfação com o uso do DIU e cinco relativos ao conhecimento sobre o método. Nenhum estudo abordou os dois desfechos simultaneamente. Observou-se conhecimento inadequado acerca do DIU, permeado por concepções equivocadas. A satisfação das mulheres com o DIU foi maior que 80% na maioria dos estudos.

**Conclusão:**

Os achados apontam para a estigmatização do DIU e para lacunas no conhecimento acerca do método, além de evidenciar alta taxa de satisfação entre as usuárias do método.

## INTRODUÇÃO

No Brasil, mais de 80% das mulheres utilizam algum método contraceptivo (MC)^(1,2)^. No entanto, mantêm-se altos os níveis de gestações não intencionais no país. A pesquisa Nascer no Brasil, realizada com mais de 20.000 mulheres no período de 2011 a 2012, apresentou uma prevalência de 55,4% de gestações não intencionais^(3)^. Em estudo mais recente realizado em oito hospitais universitários, das cinco regiões do Brasil, que avaliou 1.120 puérperas, foi identificada uma proporção de gestação não intencional de 67,4%^(4)^. Essas gestações estão associadas ao aumento da morbimortalidade materno-infantil^(5)^ e poderiam ser evitadas com o maior acesso à informação e uso consistente do MC de escolha.

A ocorrência de uma gravidez não intencional pode decorrer de uma vulnerabilidade contraceptiva favorecida pela alta taxa de descontinuidade, a depender do MC adotado^(6)^. A descontinuidade é mais frequente entre usuárias dos MC de curta duração (SARCs) do que aquelas que usam métodos de longa duração (LARCs), de 56,8% e 17,8%, respectivamente^(7)^. Entre os LARCs, tem-se o Dispositivo Intrauterino (DIU), método reversível e com alto grau de eficácia^(8,9)^. Sua eficácia pode ser comparada com a dos métodos cirúrgicos definitivos, como laqueadura tubária e vasectomia^(10)^, e contribui positivamente para a saúde das mulheres e a saúde pública ao atuar reduzindo a incidência de gestações não intencionais^(11)^.

Em 2020, o DIU foi um método utilizado por mais de 161 milhões de mulheres em todo o mundo^(6)^. Atualmente, países como China, Egito e Tunísia apresentam as maiores taxas de uso desse MC^(6)^. Entre os anos de 1995 e 2020, a proporção de usuárias de DIU diminuiu globalmente de 22% para 17%, com o Norte da África e a Ásia Ocidental registrando o maior declínio^(6)^. Em menor escala, a América Latina também apresentou diminuição na proporção do uso do DIU^(6)^, mesmo que o uso desse método em alguns países do continente tenha alcançado níveis maiores de acesso e utilização.

O Brasil, por exemplo, apresentou um aumento do uso do método no período de 1990 e 2019, entre as mulheres em idade reprodutiva, de 1,8%^(2)^ para 4,4%^(7)^, com progressões de aumento até 2030^(12)^. Esse aumento pode decorrer de políticas públicas voltadas para o planejamento reprodutivo. Entre elas, destacam-se: o Pacto pela Saúde, regulamentado pela Portaria n*o* 399, de fevereiro de 2006, do Ministério da Saúde (MS), que contou com investimento financeiro para ampliar o cardápio de contraceptivos; a Rede Cegonha instituída, no âmbito do Sistema Único de Saúde (SUS), pela Portaria n*o* 1.459, de junho de 2011, do MS, que visou assegurar o direito ao planejamento reprodutivo; e a Portaria n*o* 3.265, de dezembro de 2017, do MS, que dispõe sobre a disponibilização gratuita do DIU de cobre para anticoncepção pós-parto normal, cesárea ou pós- abortamento imediatos nas maternidades do país.

Entretanto, o uso do DIU no Brasil permanece incipiente, mesmo com declínio de utilização do método globalmente^(6)^ e com a disponibilidade prevista no SUS^(13)^. Os motivos para o não uso do DIU têm sido descritos e perpassam diversos fatores, organizacionais e individuais. Entre os fatores organizacionais, estão a oferta insuficiente do método, a aplicação recorrente de critérios que não se caracterizam como contraindicações do método^(14)^, o conhecimento inadequado dos profissionais de saúde acerca do DIU^(14,15)^, e a centralização da inserção no profissional médico^(14)^, apesar da resolução do MS, por meio da Nota Técnica nº 31/2023, dispor sobre a possibilidade de o DIU ser inserido por médicos(as) e enfermeiros(as), desde que qualificados(as) para tal^(13)^.

Entre os fatores individuais para o não uso do DIU, destaca- se o baixo conhecimento sobre fatores intrínsecos ao método, como sua eficácia, efeitos colaterais, segurança e mecanismo de ação^(16)^. Vale considerar ainda as concepções equivocadas, que criam uma atmosfera de desconfiança e hesitação em relação ao método. Estudo realizado na Etiópia com mulheres que inseriram o DIU no pós-parto encontrou que mulheres com maior conhecimento acerca do DIU tinham quase quatro vezes mais chance de utilizar o método, quando comparadas àquelas com menor conhecimento^(17)^.

Ademais, o conhecimento adequado acerca do método pode impactar positivamente na satisfação com o uso, pois pressupõe-se que a usuária conseguirá optar pelo método que mais se adeque às suas necessidades e expectativas^(18)^. Ainda, a satisfação com o método adotado está relacionada a uma maior continuidade do uso^(19)^ e, consequentemente, a uma diminuição da vulnerabilidade contraceptiva^(6)^. A satisfação com o uso do DIU é elevada no âmbito mundial. Estudo realizado com mulheres de 11 países europeus apontou que 78% e 76% estavam muito satisfeitas com o DIU hormonal e de cobre, respectivamente^(20)^. Vale ressaltar, entretanto, que apesar da presença de mitos e conhecimento inadequado por parte da população egípcia^(21)^ em relação ao DIU, as usuárias reportaram alta satisfação (77,8%)^(22)^, e o país se mantém entre aqueles cujas mulheres mais utilizam o método^(6)^.

Diante desses dados, percebem-se a importância de sintetizar as evidências relacionadas ao conhecimento acerca do DIU e a satisfação com o uso do método no Brasil para identificar possíveis características da população brasileira que possam justificar a menor utilização do método no país. Além disso, sintetizar essas evidências poderia contribuir para melhor entendimento desses aspectos fundamentais em um contexto de subutilização do método, menor que 5%, no Brasil. A síntese proposta poderia ainda contribuir para o desenvolvimento de ações de melhoria do acesso e do uso correto do método. Assim, esta revisão sistemática tem como objetivo identificar o conhecimento de mulheres sobre o DIU e a satisfação das usuárias com o método no Brasil.

## MÉTODO

### Tipo de Estudo

Trata-se de revisão sistemática desenvolvida para responder à pergunta “Qual o conhecimento de mulheres sobre o DIU e a satisfação das usuárias do método no Brasil?”. As etapas para o relato da revisão sistemática seguiram as diretrizes do *Preferred Reporting Items for Systematic reviews and Meta-Analyses* (PRISMA)^(23)^: 1) identificação do tema e seleção da hipótese ou questão de pesquisa; 2) estabelecimento de critérios para inclusão e exclusão de estudos; 3) definição das informações a serem extraídas dos estudos selecionados; 4) avaliação dos estudos incluídos na revisão; 5) interpretação dos resultados; e 6) apresentação da revisão/síntese do conhecimento. Este estudo também seguiu as diretrizes *Sex and Gender Equity in Research* (SAGER)^(24)^, que dispõem sobre o uso cauteloso do sexo e gênero em publicações científicas e a padronização dessas definições. Neste estudo, incluímos como categoria de busca mulheres cisgênero (designadas com o sexo feminino ao nascer e que se identificam como mulheres), homens transgênero (designados com o sexo feminino ao nascer, mas que se identificam como homens) e pessoas não binárias com útero (designadas com o sexo feminino ao nascer, mas que não se identificam nem como homens nem como mulheres).

### Critérios de Elegibilidade

Foram incluídos estudos primários em inglês, espanhol, português e francês, que estivessem disponíveis na íntegra, sem restrição de tempo de publicação, com abordagem metodológica qualitativa e/ou quantitativa que avaliaram a satisfação ou conhecimento sobre o DIU de pessoas com útero (mulheres cisgênero, homens transgênero, e pessoas não binárias com útero), independentemente da idade e que viviam no Brasil. Nos estudos da satisfação, o uso atual ou passado do DIU como MC foi obrigatório. Em contrapartida, foram considerados elegíveis artigos que investigavam o conhecimento sobre o DIU tanto em usuários quanto em não usuários do método.

Foram excluídos estudos que consideravam “conhecimento” como puramente saber da existência do DIU enquanto um MC ou que estudavam o conhecimento de profissionais de saúde sobre o método. Revisões sistemáticas, relatos de caso, resenhas, editoriais e documentos ministeriais também foram excluídos.

### Registro do Protocolo

O protocolo contendo as etapas da revisão e da síntese dos achados foi registrado e publicado no *International Prospective Register of Systematic Reviews* (PROSPERO) (http://www.crd.york.ac.uk/PROSPERO), sob o número CRD42024611232.

### Estratégia de Busca

Foi utilizado o mnemônico PECO (P: população/pacientes; E: exposição; C: comparação/controle; O: *outcome*/desfecho)^(25)^. Neste estudo, a população consistiu em pessoas com útero; a exposição foi o uso ou a avaliação do DIU; e os desfechos foram a satisfação e o conhecimento em relação ao método. A estratégia de busca foi elaborada utilizando descritores e seus termos alternativos retirados dos sistemas de classificação de termos para indexação: Descritores em Ciência da Saúde (DeCS), utilizando-se quatro idiomas (português, inglês, espanhol e francês); e o *Medical Subject Headings* (MeSH), para a busca em inglês.

As bases de dados utilizadas na busca bibliográfica foram PubMed, Scopus, Web of Science (WoS) e LILACS via Biblioteca Virtual em Saúde (BVS). A busca nas bases Scopus e WoS foi realizada por meio do consórcio do portal de periódicos da Coordenação de Aperfeiçoamento de Pessoal de Nível Superior (CAPES). A busca foi realizada em junho de 2023, sem restrições de data de publicação ou demais filtros.

Os descritores foram combinados pelos operadores booleanos (*OR* e *AND*), respeitando-se as características de busca em cada base. A busca gratuita na WoS permite no máximo 50 termos. Por isso, a estratégia foi reduzida com a utilização do “*” para termos com variações. Ainda, realizou-se busca adicional nas listas de referências dos estudos incluídos na revisão. A estratégia de busca utilizada na BVS contou com termos em português, inglês, espanhol e francês. Já nas demais bases de dados, apenas os termos em inglês foram utilizados. A seguir está descrita a estratégia de busca: (Mulher *OR* Mulheres *OR* Meninas *OR* Adolescente *OR* Adolescentes *OR* Jovens *OR* Jovem *OR Women OR Girls OR Girl OR Woman OR Adolescent OR Adolescents OR Teens OR Teen OR Teenagers OR Teenager OR Mujeres OR Femmes OR* “Pessoas Transgênero” *OR* “Homem Transexual” *OR* “Homens Trans” *OR* “*Transgender Persons” OR “Transgender Man” OR “Trans Man” OR “Transgender Men” OR “Personas Transgénero” OR “Personnes transgenres” OR “Pessoas Não-binárias” OR “Non-binary People” OR “Non-binary Person” OR “Personas No Binarias” OR “Personnes Non Binaires”) AND (“Dispositivos Intrauterinos” OR “Anticoncepcionais Intrauterinos” OR* DIU *OR* “Sistema Intrauterino” *OR SIU OR* “DIU Hormonal” *OR* “DIU de Cobre” *OR “Intrauterine System” OR IUS OR “Intrauterine Devices” OR “Device, Intrauterine” OR “Devices, Intrauterine” OR “Intrauterine Device” OR “Contraceptive IUDs” OR “Contraceptive IUD” OR “IUD, Contraceptive” OR “IUDs, Contraceptive” OR “Contraceptive Devices, Intrauterine” OR “Contraceptive Device, Intrauterine” OR “Device, Intrauterine Contraceptive” OR “Devices, Intrauterine Contraceptive” OR “Intrauterine Contraceptive Device” OR “Intrauterine Contraceptive Devices” OR “Unmedicated IUDs” OR “Unmedicated IUD” OR “IUD, Unmedicated” OR “Intrauterine Devices, Medicated” OR “Device, Medicated Intrauterine” OR “Devices, Medicated Intrauterine” OR “Intrauterine Device, Medicated” OR “Medicated Intrauterine Device” OR “Intrauterine Devices, Hormone-Releasing” OR “Device, Hormone-Releasing Intrauterine” OR “Devices, Hormone-Releasing Intrauterine” OR “Hormone-Releasing Intrauterine Device” OR “Hormone-Releasing Intrauterine Devices” OR “Intrauterine Device, Hormone-Releasing” OR “Intrauterine Devices, Hormone Releasing” OR “IUD, Hormone Releasing” OR “Hormone Releasing IUD” OR “Medicated Intrauterine Devices” OR “Hormone-Releasing IUDs” OR “Hormone-Releasing IUD” OR “IUD, Hormone-Releasing” OR “IUDs, Hormone-Releasing” OR “Intrauterine Devices, Copper” OR “Copper Intrauterine Device” OR “Device, Copper Intrauterine” OR “Devices, Copper Intrauterine” OR “Intrauterine Device, Copper” OR “Copper-Releasing IUDs” OR “Copper-Releasing IUD” OR “IUD, Copper-Releasing” OR “IUDs, Copper-Releasing” OR “IUD, Copper Releasing” OR “Copper Releasing IUD” OR “Copper Intrauterine Devices” OR “Dispositifs Intra-utérins” OR IUD OR “Système Intra-utérin” OR* “Métodos Contraceptivos” *OR “MC” OR “Contraceptive Device” OR Contraception OR “Contraceptive Methods” OR “Contraceptive Method” OR* “Métodos Anticonceptivos” *OR “Méthodes Contraceptives*”) *AND* (“Satisfação do Paciente” OR Satisfação *OR “Patient Satisfaction” OR Satisfaction OR “Satisfacción del Paciente” OR “Satisfaction des Patients” OR* Conhecimento *OR Conhecimentos OR Knowledge OR Conocimiento OR Savoir*) *AND* (Brasil *OR Brazil OR Brésil*).

### Seleção dos Estudos

A busca foi realizada em junho de 2023, e recuperou 1.215 artigos. Os estudos encontrados foram exportados para a plataforma Rayyan - plataforma *online* gratuita, utilizada para gerenciar artigos na construção de revisões sistemáticas, revisões integrativas e outros tipos de sínteses de evidências – para exclusão de duplicatas e seleção dos estudos em três etapas. A primeira etapa do processo foi conduzida por duas revisoras independentes, C.O.G.L.S. e S.N.R, que realizaram a leitura dos títulos e resumos de cada artigo para a seleção dos estudos de acordo com os critérios pré-estabelecidos. Desacordos entre estas revisoras (n = 26) foram desempatados por uma terceira revisora (M.S.F.M.). Finalmente, os artigos selecionados foram lidos na íntegra para a inclusão final.

Após a leitura completa, os títulos dos estudos selecionados foram buscados no site *Retraction Watch* (https://retractionwatch.com/), que identifica artigos retratados que se encontram disponíveis em bases de dados como Scopus, *National Library of Medicine*, entre outras. Além disso, foi realizada a investigação das revistas nas quais foram publicados os estudos selecionados no *Beall’s List* (https://beallslist.net), que identifica revistas e editoras com práticas predatórias.

### Extração de Dados

Para a extração de dados, foi elaborada uma planilha no *software Excel/Microsoft*, e o processo foi conduzido novamente por duas revisoras, C.O.G.L.S. e S.N.R., de forma independente. As seguintes informações foram extraídas dos estudos selecionados: 1) características relacionadas ao estudo: título, autores, ano de publicação, idioma, local, revista de publicação e momento da coleta; 2) atributos metodológicos: objetivos, tipo de estudo, tamanho da amostra, forma de coleta de dados e população; 3) características da população: faixa etária e se são usuárias ou não do método; e 4) desfechos de interesse: conhecimento das mulheres acerca do DIU e a satisfação das usuárias com o método.

Os achados das duas revisoras foram comparados a fim de identificar possíveis discrepâncias e avaliar a consistência das informações coletadas. As discordâncias entre os resultados foram resolvidas por consenso.

### Análise de Dados

A avaliação da qualidade metodológica dos estudos selecionados foi realizada por meio de critérios do JBI para verificação do risco de viés e como o mesmo foi abordado pelos autores no estudo^(26)^. Os estudos quantitativos foram avaliados por meio do questionário para pesquisas de prevalência/incidência, que contém nove questões; e os estudos qualitativos foram avaliados por meio de *checklist* para estudos qualitativos, com dez questões. Em ambos os instrumentos, para cada pergunta, pode-se responder “sim”, “não”, “não está claro” ou “não se aplica”^(26)^. Quanto mais “sim” o estudo receber, maior é a pontuação, o que indica menor risco de viés.

Os estudos de prevalência que receberam “sim” para sete ou mais questões foram classificados com alta qualidade metodológica; os que receberam “sim” para cinco a seis das questões foram classificados com média qualidade metodológica; e os estudos que receberam “sim” para quatro ou menos foram classificados com baixa qualidade metodológica. Em contrapartida, os estudos qualitativos que receberam “sim” para oito ou mais questões foram classificados com alta qualidade metodológica; os que receberam “sim” para cinco a sete das questões foram classificados com média qualidade metodológica; e os estudos que receberam “sim” para quatro ou menos foram classificados com baixa qualidade metodológica. A avaliação foi feita por duas revisoras, C.O.G.L.S. e S.N.R., de forma independente. Os resultados foram comparados e as discordâncias foram resolvidas por consenso.

Quanto à análise dos dados extraídos, realizou-se uma síntese qualitativa. Consideraram-se os atributos metodológicos dos estudos, o desfecho mensurado e os principais resultados levantados para a criação de categorias maiores (conhecimento e satisfação) e categorias menores (instrumentos para mensurar o conhecimento e motivos relacionados à satisfação ou insatisfação), de acordo com a recorrência dos achados. Essa categorização decorreu da análise temática^(27)^ dos artigos incluídos na revisão.

## RESULTADOS

### Seleção dos Estudos

Dos 1.215 estudos encontrados, 490 foram excluídos por duplicidade, restando 725 artigos (Figura 1). Após a leitura de títulos e resumos, 671 estudos foram excluídos, pois não atenderam aos critérios de inclusão, restando 54 artigos. Após a leitura completa, 42 estudos foram excluídos por também não satisfazerem os critérios de inclusão, a saber: 11 não discutiram sobre o DIU, mas, sim, outros MC; 18 investigaram se a população estudada apenas sabia da existência do DIU; 3 não atendiam aos critérios de inclusão estabelecidos para a população deste estudo (um mensurando os desfechos em profissionais de saúde, um com amostra de pessoas não brasileiras e um com população de homens cisgênero); 3 estavam duplicados, escritos em português e em inglês; 4 eram dissertações (3) ou teses (1), cujos artigos já haviam sido publicados; 2 não foram encontrados na íntegra gratuitamente; e 1 artigo estudava apenas os motivos de escolha do DIU como MC e não abordava os desfechos de interesse da presente revisão. Assim, foram incluídos 12 artigos nesta revisão. Nenhum dos artigos selecionados foi retratado, e nenhuma das revistas de publicação foi considerada predatória.

**Figura 1:** Fluxograma de seleção dos estudos para a revisão – Brasil, 2024.

Das produções científicas encontradas, cinco discutiram aspectos relacionados ao conhecimento sobre o método (Tabelas 1 e 2), enquanto os outros sete estudaram a satisfação das usuárias com o DIU (Tabela 3). Nenhum artigo avaliou os dois desfechos concomitantemente e nem teve como população de estudo homens trans ou pessoas não binárias. Assim, o presente estudo discutirá apenas resultados encontrados em populações de mulheres cisgênero e utilizará, então, pronomes femininos para se referir aos achados.

**Tabela 1:** Características dos estudos sobre o conhecimento das mulheres acerca do Dispositivo Intrauterino – Brasil, 2024.

Primeiro autor/ano e número de citação	População/amostra	Uso de DIU	Abordagem metodológica e tipo de estudo	Local	JBI**
Camiá/1997(^28^)	100 puérperas	Não	Qualitativo com análise de discurso	Campo Grande, MS	6/10
Espejo/2003(^31^)	472 mulheres entre 30 e 49 anos	Não	Quantitativo transversal	Campinas, SP	9/9
Silva-Filho/2016(^30^)	380 mulheres entre 20 e 30 anos	s/e*	Quantitativo transversal	Brasil	4/9
Diniz/2020(^29^)	22 mulheres entre 14 e 24 anos	Não	Qualitativo com análise temática	São Luís, MA, Balsas, MA, e Santana do Ipanema, AL	7/10
Borges/2020(^16^)	1.858 mulheres de 18–49 anos	s/e*	Quantitativo transversal	São Paulo, SP, Aracaju, SE e Cuiabá, MT	8/9

Nota: *: Sem especificação;

**: Verificação do risco de viés por meio de critérios JBI; DIU: Dispositivo Intrauterino; MS: Mato Grosso do Sul; SP: São Paulo; MA: Maranhão; AL: Alagoas; SE: Sergipe; MT: Mato Grosso.

**Tabela 2:** Conhecimento sobre o Dispositivo Intrauterino entre as mulheres brasileiras usuárias ou não do método contraceptivo. Brasil, 2024.

Primer autor/año y no citación	Objetivo	Instrumento	Preguntas	Resultados principales
Camiá/1997(^28^)	Avaliar o grau de informações de puérperas quanto aos métodos contraceptivos	Questionário estruturado com 10 questões abertas e fechadas. Nenhuma questão específica sobre o DIU, e ele poderia ser mencionado livremente.	Você ouviu falar como evita gravidez? (sim/não); O que você ouviu falar para evitar gravidez? (aberta); O que você sabe? (aberta para falar sobre os métodos citados).	Correto: 19,4%* Parcialmente correto: 41,9%* Incorreto: 38,7%*
Espejo/2003(^31^)	Avaliar o conhecimento sobre métodos anticoncepcionais e a sua possível associação com as características socioeconômicas e demográficas.	Questionário estruturado com 18 questões, sendo 6 afirmativas sobre métodos contraceptivos e, destas, apenas 1 sobre o DIU.	Quando uma mulher usa o DIU e fica grávida, o bebê nasce com o DIU grudado nele. (Fechada - concordo, discordo ou mais ou menos).	Responderam que, em caso de falha, o bebê nascerá com o DIU grudado nele (sem porcentagem descritas).
Silva-Filho/ 2016(30)	Criar dados do conhecimento de mulheres latino-americanas sobre o DIU.	Survey online com 8 afirmativas sobre o DIU com opções de resposta “sim” ou “não”.	1 – Já escutei histórias negativas sobre o DIU na TV, revistas ou jornais.	Cerca de 30% relataram terem escutado histórias negativas sobre o DIU de amigos e familiares.
			2 – Já escutei histórias negativas sobre o DIU na internet.	Cerca de 10% relataram terem escutado histórias negativas sobre o DIU de profissionais de saúde.
			3 – Já escutou histórias negativas sobre o DIU de amigos e familiares.	Cerca de 25% acreditam que o DIU não pode ser utilizado por nulíparas.
			4 – Já escutou histórias negativas sobre o DIU de profissionais da saúde.	Cerca de 15% das mulheres concordaram que o DIU é mais eficaz que outros métodos contraceptivos
			5 – Só pode ser utilizado por mulheres que já tiveram filhos.	Cerca de 40% concordaram que se trata de um método mais caro do que a maioria dos outros métodos contraceptivos.
			6 – Só pode ser usado por mulheres mais velhas (40+).	Os outros resultados possuíam valor p > 0,05.
			7 – É mais eficaz que outros métodos contraceptivos	
			8 – É mais caro que a maioria dos outros métodos contraceptivos.	
Diniz/2020(29)	Descrever a percepção e necessidades de mulheres jovens que buscam serviços de saúde sexual e reprodutiva.	Entrevista semiestruturada que seguiu um guia de tópicos**.	Levantadas questões sobre o Zika vírus e sua transmissão sexual, além de contracepção, aborto, gestação e necessidades em saúde sexual e reprodutiva.	As mulheres relataram medo da inserção por terem ouvido histórias em suas comunidades.
				Associaram ao uso do DIU: câncer, aborto, doença inflamatória pélvica, infertilidade e dor;
				Ainda, as mulheres referiram medo de inserir um objeto estranho no corpo.
Borges/2020(16)	Analisar o nível de conhecimento sobre o DIU entre mulheres em idade reprodutiva.	Questionário estruturado com 6 afirmativas sobre o DIU com opções de resposta “concordo”, “discordo”, “não sei”.	1 – O homem sente o DIU durante a relação sexual.	1 – 53,7% não sabiam responder se o homem sente o DIU durante a relação sexual e 4,9% concordavam.
			2 – O DIU é colocado por meio de uma cirurgia.	2 – 24,3% acreditavam que o DIU é inserido por meio de cirurgia e 26,1% não sabiam responder.
			3 – O DIU é abortivo.	3 – 13,7% concordaram que o DIU é abortivo e 32,2% não sabiam responder.
			4 – Depois de retirado o DIU, a mulher tem dificuldade para engravidar.	4 – 13,3% concordaram que a mulher terá dificuldade para engravidar após retirar o DIU e 33,8% não souberam responder.
			5 – O DIU aumenta o risco de câncer de útero.	5 – 21% acreditavam que o DIU aumenta o risco de câncer de útero e 41,4% não sabiam responder.
			6 – O DIU provoca muitos efeitos colaterais desagradáveis.	6 – 31,3% concordaram que o DIU provoca muitos efeitos colaterais desagradáveis e 39,8% não sabiam responder.
				Observação: 7,2% das mulheres acertaram todos os seis itens e 15,3% erraram todos. A mediana de acertos foi igual a 3.

Nota: DIU: Dispositivo Intrauterino; n^º^: número; MC: métodos contraceptivos;

*: Classificação extraída do artigo de origem;

**: Não foram descritas no artigo original as perguntas realizadas de acordo com cada tópico.

**Tabela 3:** Satisfação com o uso do Dispositivo Intrauterino pelas mulheres brasileiras usuárias do método – Brasil, 2024.

Primeiro autor/ano e número citação	População/amostra	Abordagem metodológica e tipo de estudo	Local	Instrumento	Resultado principal	JBI**
Albuquerque/ 2021(^32^)	158 mulheres no pós-parto	Quantitativo coorte prospectiva	Fortaleza, CE	Questionário com escalas Likert	6sem: 92,4% satisfeitas 6m: 86,9% satisfeitas	6/9
Scavuzzi/2016(^33^)	157 nuligestas e mulheres com parto anterior	Quantitativo transversal	Recife, PE	Questionário estruturado com escala 0–10 de satisfação	Nulíparas: 77,8% completamente satisfeitas C/ parto anterior: 76,7% completamente satisfeitas	6/9
Laporte/2022(^38^)	517 mulheres usuárias do DIU hormonal	Quantitativo transversal	Campinas, SP	Questionário fechado	91% das mulheres satisfeitas com o DIU 97% recomendariam para outras mulheres	4/9
Oliveira/2023(^34^)	100 mulheres entre 15 e 24 anos	Quantitativo coorte prospectiva	Belo Horizonte, MG	Questionário estruturado	12m: 93,9% satisfeitas 24m: 100% satisfeitas 36m: 96,8% satisfeitas	5/9
Carvalho/2017(^35^)	251 mulheres usuárias do DIU hormonal entre 18 e 45 anos	Quantitativo Coorte Prospectiva	Campinas, SP	Questionário estruturado	12m: 93,1% muito satisfeitas ou satisfeitas*	5/9
Giovanelli/2021(^36^)	294 adolescentes com <19anos	Quantitativo Coorte Prospectiva	São Bernardo do Campo, SP	Questionário estruturado	12m: 85,7% satisfeitas*	5/9
Borges/2017(^37^)	668 mulheres de 18–49 anos	Quantitativo transversal	São Paulo, SP	Questionário estruturado	Usuárias do DIU: 94,7% satisfeitas	9/9

Nota: m: Meses; sem: Semanas; MC: métodos contraceptivos;

*: Apesar do estudo ser definido pelos autores como uma coorte prospectiva, o desfecho de interesse desta revisão foi mensurado em apenas um tempo.

**: Verificação do risco de viés por meio de critérios JBI.

### Conhecimento

Os cinco estudos sobre o conhecimento das mulheres sobre o DIU eram datados de 1997 a 2020, e apenas dois foram publicados nos últimos cinco anos (Tabela 1). Em relação à abordagem metodológica, três são quantitativos transversais, e dois qualitativos, um com análise temática e outro com análise de discurso. Os estudos investigaram o conhecimento em não usuárias do método (n = 3) e em mulheres que usavam ou não o DIU (n = 2).

O tamanho amostral desses estudos variou entre 22 e 1.858 mulheres. Os estudos avaliaram mulheres entre 14 e 49 anos de idade, porém cada um com uma faixa etária diferente. Apenas um estudo considerou a amostra coletada no Brasil, independentemente da região. Os demais eram estudos com amostras locais ou regionais, como interestaduais (n = 2) ou no âmbito de um único município (n = 2). Dos estudos realizados em municípios, um era do estado de São Paulo e um do Mato Grosso do Sul (Tabela 1).

Nos estudos que avaliaram o conhecimento sobre o DIU, as desinformações sobre o método ficaram evidentes, como as crenças de que o DIU: é abortivo^(16,28,29)^; causa câncer^(28,29)^, doença inflamatória pélvica^(29,30)^ e gravidez ectópica^(30)^; dificulta que a mulher engravide após a retirada ou causa infertilidade^(16)^; só é indicado para mulheres que já tenham tido filho(s)^(30)^; e que, em caso de falha, o bebê nascerá com o DIU grudado nele^(31)^ (Tabela 2).

As mulheres relataram terem escutado histórias negativas sobre o DIU de amigos, familiares e até de profissionais de saúde^(29,30)^. Além disso, o medo da inserção, de introduzir um objeto estranho no corpo e da dor no momento da inserção ou durante o uso, foi reportado pelas mulheres como motivos para o receio de adotar o método^(29)^. Lacunas no conhecimento sobre o local de inserção do DIU^(28)^ e sobre o que é o método, com mulheres acreditando ser uma argola ou disco, por exemplo^(28)^, foram encontradas.

Em estudo qualitativo^(28)^, categorias foram criadas pelos autores para que se considerassem as respostas como corretas, parcialmente corretas e incorretas. Neste estudo, foram consideradas corretas quando as mulheres disseram que o DIU é um aparelho colocado no útero pelo médico e que deve ser revisado periodicamente. Considerou-se parcialmente correto quando relatado que ele era inserido dentro da mulher, no colo do útero ou na vagina, tratando-se de algo similar a uma linha de pescar com duas pontas e que era abortivo. Finalmente, julgou-se incorreto quando consideravam que o DIU era similar a uma pele, que evitava a gravidez e doenças; era uma argola recoberta em pomada a ser inserida no útero; e que se tratava de um disco colocado na entrada do útero que funciona como uma tampa. Considerando tais categorias, 19,4% apresentaram conhecimento acerca do DIU correto, 41,9% parcialmente correto, e 38,7% incorreto (Tabela 2).

Pesquisa que investigou o conhecimento sobre o DIU de usuárias de unidades de saúde^(16)^ realizou seis afirmativas, para as quais as mulheres poderiam responder com “concordo”, “discordo” ou “não sei”, a saber: 1) o DIU é abortivo; 2) depois de retirado o DIU, a mulher tem dificuldade para engravidar; 3) o DIU é colocado por meio de uma cirurgia; 4) o homem sente o DIU durante a relação sexual; 5) o DIU aumenta o risco de câncer de útero; 6) o DIU provoca muitos efeitos colaterais desagradáveis. Das 1.858 mulheres que compuseram a amostra, apenas 7,2% acertaram todos os seis itens e 15,3% erraram todos, e a mediana de acerto foi igual a três (Tabela 2).

### Satisfação

Sete estudos investigaram a satisfação das usuárias com o método e foram publicados de 2016 a 2022, quatro deles nos últimos cinco anos (Tabela 3). Em relação à abordagem metodológica, todos os estudos são quantitativos, predominando estudos prospectivos (n = 4), seguidos dos transversais (n = 3). O tamanho amostral dos estudos variou entre 100 e 668 mulheres. Os estudos analisaram mulheres com idades entre 15 e 49 anos, cada um com faixas etárias distintas. Todos utilizaram amostras locais no âmbito municipal. Desses, quatro estudos foram realizados no estado de São Paulo, um em Minas Gerais, um no Ceará e um em Pernambuco.

Altas porcentagens de satisfação com o uso do DIU foram encontradas nos estudos com a população brasileira de usuárias do método, a saber: 92,4% e 86,9% estavam satisfeitas após 6 semanas e 6 meses da inserção, respectivamente^(32)^; 77,8% das nulíparas e 76,7% das mulheres com parto anterior estavam completamente satisfeitas^(33)^; 93,9%, 100% e 96,8% satisfeitas com 12, 24 e 36 meses de uso, respectivamente^(34)^; 93,1% satisfeitas aos 12 meses após inserção^(35)^; 85,7% satisfeitas aos 12 meses após parto^(36)^; 94,7%^(37)^ e 91%^(38)^ satisfeitas, independentemente do tempo de uso.

Entre os sete artigos que estudaram a satisfação das usuárias, quatro investigaram os fatores relacionados à insatisfação com o uso do DIU; dois estudos discutiram os fatores relacionados à satisfação com o método; e um estudo discutiu os motivos para satisfação e insatisfação. Os resultados foram sintetizados na Tabela 4.

**Tabela 4:** Fatores relacionados à (in)satisfação com o uso do Dispositivo Intrauterino entre as brasileiras – Brasil, 2024.

Insatisfação	Número de citação
Aumento ou prolongamento de sangramento menstrual	(32,35,36)
Dismenorreia	(32,36)
Sangramento de escape	(35)
Outros efeitos colaterais (como acne, ganho de peso e enxaqueca)	(35)
**Satisfação**	**Número de citação**
Amenorreia ou diminuição do fluxo menstrual	(35,38)
Confiança no método	(33)
Diminuição da dismenorreia	(38)
Consulta com profissional de saúde antes de utilizar o método	(37)

## DISCUSSÃO

Os achados deste estudo apontam para a estigmatização do DIU e para lacunas no conhecimento acerca deste, fatores que podem impactar no desejo de uso do método pelas mulheres no país. Destaca-se a ausência de padronização de questões ou aspectos a serem mensurados para avaliar o conhecimento acerca do método, o que em alguma medida compromete a comparação dos resultados dos estudos existentes. Entretanto, esses achados nos estudos brasileiros corroboram os de revisão sistemática realizada com estudos de outros países, nos quais as mulheres também associaram o DIU à infertilidade, à gravidez ectópica e ao câncer, além da crença do método não poder ser utilizado por nulíparas, por exemplo, principalmente nos países de baixa e média renda da Ásia e África^(39)^.

Na presente síntese, foram pontuados como motivo para o não uso do método o receio de introduzir um objeto estranho no corpo e o medo do procedimento de inserção. Tais achados corroboram estudo realizado na Coreia que investigou as percepções das mulheres acerca do DIU^(40)^. Adicionalmente, foi relatado pelas mulheres brasileiras o medo da dor durante a inserção, o que favorecia o não uso. Entretanto, estudo realizado em São Paulo com mulheres que passaram pela inserção do DIU aponta que menos de 19% classificaram a dor como intensa, e a maioria classificou a dor como moderada (44%) e leve (37,3%)^(41)^. Por outro lado, a dor é uma experiência individual e que pode estar presente na inserção do DIU, devendo o profissional informar as mulheres sobre as estratégias para mitigá-la, incluindo a oferta de analgesia^(42)^.

A falta desse conhecimento e dessa prática também pode contribuir para menor uso do método. Adicionalmente, os achados também reforçam que a falta de conhecimento e concepções errôneas sobre o método e sua inserção podem estimular medos exagerados, trazendo prejuízos às próprias mulheres por não usarem o método por essas questões. Assim, os resultados encontrados sobre o conhecimento inadequado acerca do DIU por parte das mulheres brasileiras apontam que o acesso a serviços de planejamento reprodutivo, especialmente o acesso a informações adequadas acerca desse método, parece estar aquém do preconizado^(43)^.

Apesar do baixo e equivocado conhecimento sobre o método, cabe ressaltar que as mulheres têm interesse em receber mais informações. O estudo *Thinking About Needs in Contraception*, inquérito quantitativo *online* sobre as opiniões dos profissionais de saúde e das mulheres quanto aos aspectos do aconselhamento contraceptivo e do uso de contraceptivos, identificou que 66% das mulheres estavam interessadas em receber mais informações sobre todos os métodos e que 69% consideravam utilizar LARCs se recebessem informações mais abrangentes^(44)^. Dessa forma, reitera-se que se as mulheres recebessem informações adequadas, as preocupações e receios relatados nos estudos poderiam ser atenuados.

Mesmo com a escassa avaliação sobre a fonte das desinformações acerca do DIU nos estudos, não é irreal supor que as mulheres possam ter buscado na internet respostas para suas dúvidas. Nesse contexto, é necessário considerar a não confiabilidade desses sites que, mesmo quando especializados em contracepção, propagam informações inconsistentes e equivocadas sobre o método, podendo contribuir para o não uso do mesmo^(45)^. Em contrapartida, quando as informações são fornecidas por profissionais de saúde capacitados, o interesse no uso aumenta. Estudo realizado em São Paulo encontrou que mais de 98% das mulheres que inseriram o DIU com obstetrizes e enfermeiras obstetras relataram que receberam orientação de planejamento reprodutivo antes da inserção e que, dessas, praticamente todas (98,6%) consideraram que foi suficiente para que se sentissem seguras com o uso do DIU^(41)^, mesmo que estudos desta revisão tenham mostrado que as mulheres reconhecem o profissional médico como aquele responsável pela inserção.

Enfermeiros capacitados têm respaldo para inserir o DIU^(13)^ e, no âmbito da enfermagem, têm competência privativa para atuação no planejamento familiar e reprodutivo^(46)^. Adicionalmente, estudo realizado com dados do Sistema de Informação em Saúde para a Atenção Básica no ano de de 2021 aponta que são os enfermeiros que realizam a maioria (cerca de 76%) das consultas individuais relacionadas ao DIU, enquanto os profissionais médicos realizaram aproximadamente 24% das consultas para orientação acerca do método^(47)^. Estudo conduzido na Etiópia investigou fatores associados ao uso do DIU pós-parto, e encontrou que mulheres que receberam orientação profissional após o parto tinham três vezes mais chance de utilizar o DIU do que aquelas que não receberam essa orientação^(17)^.

Dessa forma, observa-se a necessidade de o profissional de saúde orientar as mulheres com informações corretas e embasamento científico. No entanto, a literatura discorre sobre a existência de uma lacuna de conhecimento apresentada por parte desses profissionais acerca do DIU. Estudo realizado na Cidade do Cabo, na África do Sul, apontou que cerca de 14% dos profissionais acreditavam que o DIU pode migrar no corpo, 11,1% que aumenta a chance de aborto, 18,1% que está associado a gravidez ectópica e 22,2% que está associado a DIP^(15)^.

Ter acesso a conhecimentos embasados cientificamente sobre MC, especialmente por profissionais capacitados, é fundamental para que as mulheres tenham condição de tomar decisões sobre a saúde sexual e reprodutiva, permitindo que escolham o método mais adequado às suas necessidades e circunstâncias de vida^(18)^. Essas ações educativas, de aconselhamento coletivo e individual, configuram o preconizado no contexto do planejamento reprodutivo^(43)^. No entanto, estudo realizado com amostra representativa da população brasileira encontrou que 95,4% das mulheres em 2019 não tinham participado de grupos de planejamento reprodutivo^(1)^. O estudo norte-americano CHOICE mostrou que o aumento do conhecimento e a remoção de barreiras financeiras levaram a um aumento no uso, e na taxa de continuação e satisfação com contraceptivos reversíveis de longa duração^(48)^, reforçando a necessidade das orientações.

Os estudos incluídos nesta revisão também mostraram alta taxa de satisfação entre as usuárias do DIU. Entretanto, observou-se uma falta de parâmetros padronizados para avaliar o nível de satisfação das usuárias. Foram utilizados diferentes instrumentos, como escala Likert, escala 0-10 e categorizações diversas (satisfeita ou insatisfeita; muito satisfeita, satisfeita, insatisfeita ou muito insatisfeita; e completamente satisfeita, parcialmente satisfeita ou insatisfeita), à semelhança do que foi observado para a avaliação do conhecimento sobre o método. Tal fato compromete, em alguma medida, a comparabilidade entre os estudos dos seus achados.

Entre os efeitos colaterais, a mudança no padrão de sangramento foi o mais citado como determinante da satisfação com o uso do DIU. O aumento do sangramento, em frequência ou intensidade, foi motivo de insatisfação, enquanto a diminuição foi fator positivo para a satisfação. Nesse sentido, mais uma vez, o conhecimento prévio poderia auxiliar as mulheres na identificação e no manejo desses efeitos. Entretanto, tais mudanças no padrão do sangramento não foram suficientes para uma diminuição da taxa de satisfação das mulheres brasileiras com o uso do DIU, como evidenciado pelas pesquisas realizadas no Brasil que apontaram, em sua maioria, mais de 80% de satisfação. Estas taxas são, inclusive, superiores às encontradas na Europa, com 76–78% muito satisfeitas e no Egito, 77,8% satisfeitas^(22)^.

Observa-se, portanto, que o DIU é um método do tipo LARCs amplamente utilizado em países de alta renda, porém pouco utilizado no Brasil. O conhecimento sobre o método pelas brasileiras é baixo e cercado de concepções equivocadas, realidade também percebida no mundo de maneira geral, o que poderia justificar em parte a subutilização do método no Brasil em comparação ao contexto mundial. Por outro lado, quando utilizam, o grau de satisfação é altíssimo, maior ainda que em outros países. A partir dos achados do presente estudo, pode-se inferir a existência de problemas no acesso, o que inclui acesso a informações, que podem estar aquém do necessário, para além da disponibilização do método no cardápio do SUS.

Considerando o interesse das mulheres em receber informações mais abrangentes sobre o método^(44)^ e os impactos dessa orientação na chance de uso^(17)^, os profissionais de saúde no Brasil podem exercer um papel chave na garantia do acesso ao conhecimento adequado. Nesse sentido, a capacitação dos profissionais também parece ser necessária, e a participação das enfermeiras e enfermeiras obstétricas tem sido fundamental para a garantia do direito e acesso a esse e outros métodos^(41,47)^. Dessa forma, espera-se que, após consulta com profissional capacitado, o nível de conhecimento das mulheres aumente, aumentando, também, as chances de utilização do DIU. Entretanto, estudo com avaliação do conhecimento das mulheres brasileiras acerca do método antes e após consulta com profissional de saúde ou intervenção educativa não foi encontrado na literatura.

Como limitação do estudo, tem-se que as evidências devem ser generalizadas com cautela, devido aos diferentes desenhos e métodos para mensuração do conhecimento sobre o método e da satisfação com ele entre as usuárias. Ademais, a produção nacional acerca do tema tem uma concentração na cidade de São Paulo, com cinco (41,67%) dos doze estudos incluídos realizados lá. Trata-se de cidade pertencente ao estado mais desenvolvido do país e cuja população poderia ter diferenças consideráveis em relação à dos demais estados do país, principalmente quando se consideram as desigualdades de acesso à contracepção previamente descritas^(1,2)^. Isso resulta em escolhas e usos diferenciados dos MC disponíveis pela população.

Ademais, nenhum estudo investigou os desfechos em homens transgênero ou pessoas não binárias. O acesso dessa população é ainda mais restrito do que para mulheres cisgênero, que também tem sido limitado. Estudo aponta que grupos marginalizados possuem maiores dificuldades de acesso a serviços de saúde sexual e reprodutiva^(49)^. Assim, não é irreal inferir que esse grupo tenha um menor acesso a informações e, portanto, conhecimento ainda inferior aos encontrados nos estudos incluídos nesta revisão, o que pode resultar em uma menor chance de uso por parte de homens trans e pessoas não binárias. Dessa forma, estudos que avaliem o conhecimento nessa população são necessários, além de estratégias que sejam inclusivas para garantia do uso.

Ainda, uma revisão sistemática depende da qualidade dos estudos incluídos. Na análise da qualidade metodológica dos estudos, 8 estudos foram classificados com média qualidade, principalmente pela ausência de discussão das taxas de respostas (os autores deveriam discutir claramente a taxa de resposta e quaisquer razões para a não resposta e comparar as pessoas no estudo com aquelas que não estão no estudo), além da utilização de diferentes medidas para o conhecimento e diferentes escalas para a satisfação. Portanto, evidencia-se a necessidade de que futuros estudos sejam fortalecidos metodologicamente e que permitam maior comparabilidade entre si.

Por outro lado, este estudo avança ao identificar lacunas na literatura sobre a avaliação do conhecimento das mulheres em relação ao DIU. Historicamente, os estudos focaram nos mitos acerca do DIU e mensuraram aspectos relacionados à crença das mulheres, sem padronização dos achados. Há 30 anos, o desconhecimento sobre o DIU incluía a aparência física do método, com mulheres acreditando ser uma argola ou disco, mas essas concepções não foram mais abordadas, podendo ser diferentes no dia de hoje. Pesquisas de cerca de 10 anos atrás apontaram as crenças de que o DIU é indicado apenas para mulheres que tiveram filhos, pode causar gravidez ectópica ou doença inflamatória pélvica.

A crença de que o DIU é abortivo e causa câncer, foi descrita por estudos realizados tanto em 1997 quanto em 2020, o que indica uma manutenção dessa concepção com o decorrer do tempo. Por outro lado, informações sobre o conhecimento dos efeitos colaterais, duração, eficácia e mecanismo de ação não foram contemplados, independentemente do recorte temporal. Estudos atualizados sobre concepções, conhecimento e satisfação de pessoas com útero que utilizam o DIU são necessários diante: dessas lacunas identificadas; de evidências científicas mais antigas e da persistência da falta de informações sobre o método; das consequências do (não) uso de contraceptivos, mesmo quando a pessoa não deseja engravidar; e da baixa prevalência do uso do método no Brasil.

Ainda, ao sistematizar as evidências sobre o conhecimento e satisfação com o método das brasileiras, o estudo avança podendo fornecer subsídios para o desenvolvimento de ações de melhoria do acesso ao método - que parece envolver entraves para além do conhecimento deficitário. Tal realidade deve ser pensada, especialmente no Brasil, um país de território continental, com desigualdades sociais históricas e de grande diversidade cultural e religiosa, fatores que podem ter relação com a subutilização do DIU no país.

## CONCLUSÃO

Esta revisão permitiu a síntese das evidências científicas sobre o conhecimento de mulheres brasileiras acerca do DIU e a satisfação das usuárias desse método. Os achados indicam lacunas no conhecimento das mulheres brasileiras sobre o DIU, evidenciando concepções equivocadas e mitos que o permeiam. O estudo aponta para a estigmatização do método e para a disseminação de crenças errôneas, como associações com infertilidade e câncer, inclusive por profissionais. Esses achados ressaltam a necessidade de fortalecimento e aprimoramento da orientação por parte dos profissionais de saúde, incluindo a capacitação dos mesmos e visando a adequação do conhecimento a partir da promoção ao acesso a informações precisas e embasadas cientificamente. Essa orientação deve ser realizada no contexto das ações de planejamento reprodutivo preconizadas e que também precisam ser fortalecidas.

Apesar dessa lacuna, as mulheres que utilizaram o método relataram altas taxas de satisfação, mesmo com as preocupações e desinformações prévias. Ainda que haja variações nos instrumentos de avaliação da satisfação, os resultados sugerem que uma vez superadas as barreiras iniciais, o DIU é amplamente aceito pelas mulheres brasileiras. Em última análise, a garantia de acesso a informações precisas é fundamental para garantir às mulheres uma tomada de decisão livre e informada sobre sua saúde reprodutiva, promovendo uma maior satisfação e continuidade no uso.

## Data Availability

Todo o conjunto de dados que dá suporte aos resultados deste estudo foi publicado no próprio artigo.

# Translation: Conocimiento de las mujeres sobre el Dispositivo Intrauterino y satisfacción de los usuarios en Brasil: revisión sistemática

**DOI:** 10.1590/1980-220X-REEUSP-2024-0262es

**Authors:** Clarissa Oliveira Guimarães Lopes de Sousa, Síntia Nascimento dos Reis, Gisele Nepomuceno de Andrade, Bruna Nicole Soares dos Santos, Mariana Santos Felisbino-Mendes

**Affiliations:** 1 Universidade Federal de Minas Gerais, Escola de Enfermagem, Programa de Pós-Graduação em Enfermagem, Belo Horizonte, MG, Brasil.; 2 Universidade Federal de Minas Gerais, Escola de Enfermagem, Departamento de Enfermagem Materno-Infantil e Saúde Pública, Belo Horizonte, MG, Brasil.

**Keywords:** Conocimiento, Satisfacción del Paciente, Dispositivo Intrauterino, Revisión Sistemática, Salud de la Mujer

## Abstract

**Objetivo:**

Identificar el conocimiento de las mujeres sobre el Dispositivo Intrauterino (DIU) y la satisfacción de los usuarios con el método en Brasil.

**Método:**

Revisión sistemática, con búsqueda realizada en las bases de datos bibliográficas PubMed, Scopus, Web of Science y LILACS a través del Portal Regional de la Biblioteca Virtual en Salud en junio/2023. La investigación siguió las directrices de los Preferred Reporting Items for Systematic reviews and Meta-Analyses, y el riesgo de sesgo se midió según los criterios del JBI. Los estudios fueron seleccionados sin restricciones de tiempo de publicación, con enfoque metodológico cualitativo o cuantitativo, evaluando la satisfacción y el conocimiento de las mujeres brasileñas sobre el DIU.

**Resultados:**

Se identificaron 1.215 artículos; de estos, 12 cumplieron con los criterios finales de inclusión, que datan del período 1997–2023, siete con respecto a la satisfacción con el uso del DIU y cinco con respecto al conocimiento sobre el método. Ningún estudio abordó ambos resultados simultáneamente. Se observó un conocimiento inadecuado sobre el DIU, permeado por conceptos erróneos. La satisfacción de las mujeres con el DIU fue superior al 80% en la mayoría de los estudios.

**Conclusión:**

Los hallazgos apuntan a la estigmatización del DIU y lagunas en el conocimiento sobre el método, además de mostrar un alto índice de satisfacción entre los usuarios del método.

## INTRODUCCIÓN

Más del 80% de las mujeres utilizan algún método anticonceptivo (MAC) en Brasil^(1,2)^. Sin embargo, el número de embarazos no deseados en el país sigue siendo alto. La encuesta *Nascer no Brasil* (Nacimiento en Brasil), realizada con más de 20.000 mujeres entre 2011 y 2012, mostró una prevalencia del 55,4% de embarazos no deseados^(3)^. En un estudio más reciente realizado en ocho hospitales universitarios de las cinco regiones de Brasil, que evaluó a 1.120 puérperas, se identificó una proporción de embarazos no deseados del 67,4%^(4)^. Estos embarazos se asocian a un aumento de la morbimortalidad materna e infantil^(5)^ y podrían evitarse con un mayor acceso a la información y el uso sistemático del MAC de elección.

Embarazos no deseados pueden ser el resultado de una vulnerabilidad anticonceptiva favorecida por una tasa de interrupción elevada, en función del MAC adoptado^(6)^. La descontinuación es más frecuente entre las usuarias de métodos de acción corta (SARC) que entre las que utilizan métodos de acción prolongada (Long-acting reversible contraceptives - LARC), con 56,8% y 17,8% respectivamente^(7)^. Entre los LARC se encuentra el Dispositivo Intrauterino (DIU), un método reversible con un alto grado de eficacia^(8,9)^, comparada a la de métodos quirúrgicos definitivos como la ligadura de trompas y la vasectomía^(10)^, y contribuye positivamente con la salud de la mujer y a la salud pública al reducir la incidencia de embarazos no deseados^(11)^.

En 2020, el DIU fue utilizado por más de 161 millones de mujeres globalmente^(6)^. En la actualidad, países como China, Egipto y Túnez presentan las tasas más elevadas de uso de este MAC^(6)^. Entre 1995 y 2020, la proporción de mujeres usuarias del DIU disminuyó en todo el mundo del 22% al 17%, siendo el norte de África y Asia occidental los que registraron el mayor disminución^(6)^. En menor medida, en América Latina también hubo uma diminución de la proporción de uso del DIU^(6)^, aunque el uso de este método en algunos países del continente ha alcanzado mayores niveles de acceso y utilización.

Brasil, por ejemplo, mostró un aumento en el uso del método entre 1990 y 2019, entre mujeres en edad reproductiva, de 1,8%^(2)^ a 4,4%^(7)^, con perspectivas de aumentos hasta 2030^(12)^. Esse aumento puede deberse a las políticas públicas dirigidas a la planificación reproductiva. Entre ellas están: el Pacto por la Salud, reglamentado por la Ordenanza n*o* 399, de febrero de 2006, del Ministerio de Salud (MS), que incluyó inversiones financieras para ampliar el menú de anticonceptivos; la Red Cigüeña, establecida en el Sistema Único de Salud (SUS) por la Ordenanza n*o*. 459, de junio de 2011, que tuvo como objetivo garantizar el derecho a la planificación reproductiva; y la Ordenanza N*o* 3.265, de diciembre de 2017, que prevé la disponibilidad gratuita de DIU de cobre para la anticoncepción después del parto normal, cesárea o postaborto inmediato en las maternidades de todo el país.

Sin embargo, el uso del DIU en Brasil sigue siendo incipiente, a pesar de la disminución del uso del método a nivel mundial^(6)^ y de la disponibilidad proporcionada por el SUS^(13)^. Las razones para la no utilización del DIU han sido descritas e incluyen diversos factores, tanto organizacionales como individuales. Entre los factores organizacionales están la oferta insuficiente del método, la aplicación recurrente de criterios que no son caracterizados como contraindicaciones al método^(14)^, el conocimiento inadecuado de los profesionales de salud sobre el DIU^(14,15)^ y la centralización de la inserción en el profesional médico^(14)^, a pesar de la resolución del MS, a través de la Nota Técnica n*o* 31/2023, prever la posibilidad de inserción del DIU por médicos y enfermeros, siempre que estén habilitados para hacerlo^(13)^.

Entre los factores individuales para el no uso del DIU está el bajo conocimiento sobre factores intrínsecos al método, como su eficacia, efectos colaterales, seguridad y mecanismo de acción^(16)^. También hay que tener en cuenta las ideas erróneas, que crean un clima de desconfianza y vacilación respecto al método. Un estudio realizado en Etiopía con mujeres a las que se había insertado un DIU en el periodo posparto reveló que las mujeres con más conocimientos sobre el DIU tenían casi cuatro veces más probabilidades de utilizar el método en comparación con las que tenían menos conocimientos^(17)^.

Además, un conocimiento adecuado sobre el método puede repercutir positivamente en la satisfacción con su uso, ya que se supone que la usuaria podrá elegir el método que mejor se adapte a sus necesidades y expectativas^(18)^. Además, la satisfacción con el método adoptado se relaciona con una mayor continuidad del uso^(19)^ y, consecuentemente, con una reducción de la vulnerabilidad anticonceptiva^(6)^. La satisfacción con el uso del DIU es alta en todo el mundo. Un estudio realizado en mujeres de 11 países europeos reveló que 78% y 76% estaban muy satisfechas con los DIU hormonales y de cobre, respectivamente^(20)^. Cabe señalar, sin embargo, que a pesar de los mitos y de un conocimiento inadecuado por parte de la población egipcia^(21)^ en relación con el DIU, las usuarias declararon un alto grado de satisfacción (77,8%)^(22)^, y ese país sigue figurando entre aquellos cuyas mujeres utilizan más frequentemente ese método^(6)^.

Frente a esos datos, es importante sintetizar las evidencias relacionadas al conocimiento sobre el DIU y a la satisfacción con el uso del método en Brasil, para identificar posibles características de la población brasileña que puedan justificar el menor uso del método en el país. Además, la síntesis de estas evidencias podría contribuir a una mejor comprensión de estos aspectos fundamentales en un contexto de subutilización del método, inferior al 5%, en Brasil. La síntesis propuesta también podría contribuir al desarrollo de acciones para mejorar el acceso y el uso correcto del método. Por lo tanto, esta revisión sistemática tiene como objetivo identificar el conocimiento de las mujeres sobre el DIU y su satisfacción con el método en Brasil.

## MÉTODO

### Tipo de Estudio

Se trata de una revisión sistemática diseñada para responder a la pregunta «¿Cuál es el conocimiento de las mujeres sobre el DIU y la satisfacción de las usuarias del método en Brasil?». Los pasos para el reporte de la revisión sistemática siguieron las directrices del *Preferred Reporting Items for Systematic reviews and Meta-Analyses* (PRISMA)^(23)^: 1) identificación del tema y selección de la hipótesis o pregunta de investigación; 2) establecimiento de los criterios de inclusión y exclusión de los estudios; 3) definición de las informaciones a ser extraídas de los estudios seleccionados; 4) evaluación de los estudios incluidos en la revisión; 5) interpretación de los resultados; y 6) presentación de la revisión/síntesis del conocimiento. En este estudio también se siguieron las directrices *Sex and Gender Equity in Research* (SAGER)^(24)^, que prevén el uso prudente del sexo y el género en las publicaciones científicas y la normalización de estas definiciones. En este estudio, se incluyó como categoría de búsqueda a mujeres cisgénero (a las que se les asignó el sexo femenino al nacer y que se identifican como mujeres), hombres transexuales (a los que se les asignó el sexo femenino al nacer pero que se identifican como hombres) y personas no binarias con útero (a las que se les asignó el sexo femenino al nacer pero que no se identifican ni como hombres ni como mujeres).

### Criterios de Selección

Se incluyeron estudios primarios en inglés, español, portugués y francés, que estuvieran disponibles en su totalidad, sin restricción de tiempo de publicación, con un enfoque metodológico cualitativo y/o cuantitativo que evaluaran la satisfacción o el conocimiento sobre el DIU de personas con útero (mujeres cisgénero, hombres transgénero y personas no binarias con útero), independientemente de la edad y que vivieran en Brasil. En los estudios de satisfacción, el uso actual o pasado del DIU como MAC era obligatorio. Por otro lado, se consideraron elegibles los artículos que investigaban el conocimiento sobre el DIU tanto en usuarias como en no usuarias del método.

Se excluyeron los estudios que consideraban el «conocimiento» como el mero saber sobre la existencia del DIU como MAC o que estudiaban el conocimiento de los profesionales sanitarios sobre el método. También se excluyeron las revisiones sistemáticas, los informes de casos, las revisiones, los editoriales y los documentos ministeriales.

### Registro del Protocolo

El protocolo que contiene las etapas de la revisión y la síntesis de los hallazgos fue registrado y publicado en el *Registro Prospectivo Internacional de Revisiones Sistemáticas* (PROSPERO)(http://www.crd.york.ac.uk/PROSPERO), con el número CRD42024611232.

### Estrategia de Búsqueda

Se utilizó la nemotecnia PECO (P: población/pacientes; E: exposición; C: comparación/control; O: *resultado*)^(25)^. En este estudio, la población fue formada por personas con útero; la exposición era el uso o la evaluación del DIU; y los resultados eran la satisfacción con el método y el conocimiento del mismo. La estrategia de búsqueda fue elaborada utilizando descriptores y sus términos alternativos tomados de los sistemas de clasificación de términos para indización: Descriptores en Ciencias de la Salud (DeCS), para cuatro idiomas (portugués, inglés, español y francés); y *Medical Subject Headings* (MeSH), para la búsqueda en inglés.

Las bases de datos utilizadas para la búsqueda bibliográfica fueron PubMed, Scopus, Web of Science (WoS) y LILACS a través de la Biblioteca Virtual en Salud (BVS). La búsqueda en las bases de datos Scopus y WoS fue realizada a través del consorcio del portal de revistas Coordenação de Aperfeiçoamento de Pessoal de Nível Superior (CAPES). La búsqueda se realizó en junio de 2023, sin restricciones de fecha de publicación u otros filtros.

Los descriptores fueron combinados utilizando operadores booleanos (*OR* y *AND*), respetando las características de búsqueda de cada base de datos. La búsqueda gratuita en WoS permite un máximo de 50 términos. Por este motivo, la estrategia se redujo utilizando «*» para los términos con variaciones. Se realizó una búsqueda adicional en las listas de referencias de los estudios incluidos en la revisión. La estrategia de búsqueda utilizada en la BVS incluyó términos en portugués, inglés, español y francés. En las demás bases de datos, sólo se utilizaron términos en inglés.

La estrategia de búsqueda se describe a continuación: (Woman OR Women OR Girls OR Adolescent OR Teenagers OR Youth OR Women OR Girls OR Girl OR Woman OR Adolescent OR Adolescents OR Teens OR Teenagers OR Teenager OR Mujeres OR Femmes OR “TransgenderPersons” OR “Transsexual Man” OR “Trans Men” OR “Transgender Persons” OR “Transgender Man” OR “Transgender Man” OR “Transgender Men” OR “Personas Transgénero” OR “Personnes transgenres“ OR “Non- binary” OR “Non-binary People” OR “Non-binary Person” OR “Personas No Binarias” OR “Personnes Non Binaires”) AND (“Intrauterine Devices” OR “Intrauterine Contraceptives” OR IUD OR “Intrauterine System” OR SIU OR “Hormonal IUD” OR “Copper IUD” OR “Intrauterine System” OR IUS OR “Intrauterine Devices” OR “Device, Intrauterine” OR “Devices, Intrauterine” OR “Intrauterine Device” OR “Contraceptive IUDs” OR “Contraceptive IUD” OR “IUD, Contraceptive” OR “IUDs, Contraceptive” OR “Contraceptive Devices, Intrauterine” OR “Contraceptive Device, Intrauterine” OR “Device, Intrauterine Contraceptive” OR “Devices,Intrauterine Contraceptive” OR “Intrauterine Contraceptive Device” OR “Intrauterine Contraceptive Devices” OR “Unmedicated IUDs” OR “Unmedicated IUD” OR “IUD, Unmedicated” OR “Intrauterine Devices, Medicated” OR “Device, Medicated Intrauterine” OR “Devices, Medicated Intrauterine” OR “Devices, Medicated Intrauterine” OR “Intrauterine Device, Medicated” OR “Medicated Intrauterine Device” OR “Intrauterine Devices, Hormone-Releasing” OR “Device, Hormone-Releasing Intrauterine” OR “Devices, Hormone-Releasing Intrauterine” OR “Hormone-Releasing Intrauterine Device” OR “Hormone-Releasing Intrauterine Devices” OR “Intrauterine Device, Hormone-Releasing” OR “Intrauterine Devices, Hormone Releasing” OR “IUD, Hormone Releasing” OR “Hormone Releasing IUD” OR “Medicated Intrauterine Devices” OR “Hormone-Releasing IUDs” OR “Hormone-Releasing IUD” OR “IUD, Hormone-Releasing” OR “IUDs, Hormone-Releasing” OR “Intrauterine Devices, Copper” OR “Copper Intrauterine Device” OR “Device, Copper Intrauterine” OR “Devices, Copper Intrauterine” OR “Intrauterine Device, Copper” OR “Copper-Releasing IUDs” OR “Copper-Releasing IUD” OR “IUD, Copper-Releasing” OR “IUDs, Copper-Releasing” OR “IUD, Copper Releasing” OR “Copper Releasing IUD” OR “Copper Intrauterine Devices” OR “Dispositifs Intra-utérins” OR IUD OR “Système Intra- utérin” OR “Métodos Contraceptivos” OR “MC” OR “Contraceptive Device” OR Contraception OR “Contraceptive Methods” OR “Contraceptive Method ” OR “Métodos Anticonceptivos” OR “Méthodes Contraceptives”) AND (“Satisfação doPaciente” OR Satisfaction OR “Patient Satisfaction” OR Satisfaction OR “Satisfacción del Paciente” OR “Satisfaction des Patients” OR Conhecimento OR Knowledge OR Conocimiento OR Savoir) AND (Brasi OR Brazil OR Brésil).

### Selección de Estudios

La búsqueda se realizó en junio de 2023 y recuperó 1.215 artículos. Los estudios encontrados fueron exportados a la plataforma Rayyan – plataforma *online* gratuita utilizada para la gestión de artículos en la construcción de revisiones sistemáticas, revisiones integrativas y otros tipos de síntesis de evidencias - para excluir duplicados y seleccionar los estudios en tres etapas. La primera etapa del proceso fue realizada por dos revisores independientes, C.O.G.L.S. y S.N.R., que leyeron los títulos y resúmenes de cada artículo para seleccionar los estudios según los criterios preestablecidos. Los desacuerdos entre estos revisores (n = 26) fueron resueltos por un tercer revisor (M.S.F.M.). Por último, los artículos seleccionados se leyeron en su totalidad para su inclusión final.

Tras la lectura completa, se buscaron los títulos de los estudios seleccionados en el sitio web *Retraction Watch* (https://retractionwatch.com/), que identifica los artículos *retractados* que están disponibles en bases de datos como Scopus, la *National Library of Medicine*, entre otras. Las revistas en las que se publicaron los estudios seleccionados también se investigaron en *Beall’s List* (https://beallslist.net), que identifica revistas y editoriales con prácticas depredadoras.

### Extracción de Datos

Para la extracción de datos, se creó una hoja de cálculo con el *programa Excel/Microsoft*, y el proceso fue realizado de nuevo por dos revisores, C.O.G.L.S. y S.N.R., de forma independiente. De los estudios seleccionados se extrajo la siguiente información: 1) características relacionadas con el estudio: título, autores, año de publicación, idioma, lugar, revista de publicación y momento de la recogida; 2) atributos metodológicos: objetivos, tipo de estudio, tamaño de la muestra, forma de recogida de datos y población; 3) características de la población: grupo de edad y si son usuarias o no del método; y 4) resultados de interés: conocimiento de las mujeres sobre el DIU y satisfacción de las usuarias con el método.

Los resultados de los dos revisores se compararon para identificar posibles discrepancias y evaluar la coherencia de la información recopilada. Los desacuerdos entre los resultados se resolvieron por consenso.

### Análisis de los Datos

La calidad metodológica de los estudios seleccionados se evaluó mediante los criterios del JBI para verificar el riesgo de sesgo y cómo lo abordaron los autores en el estudio^(26)^. Los estudios cuantitativos se evaluaron mediante el cuestionario para encuestas de prevalencia/incidencia, que contiene nueve preguntas; y los estudios cualitativos se evaluaron mediante la *lista de comprobación* para estudios cualitativos, con diez preguntas. En ambos instrumentos, cada pregunta puede responderse «sí», «no», «poco claro» o «no procede»^(26)^. Cuantas más respuestas «sí» recibe un estudio, mayor es su puntuación, lo que indica un menor riesgo de sesgo.

Los estudios de prevalencia que recibieron un «sí» para siete o más preguntas se clasificaron como de alta calidad metodológica; los que recibieron un «sí» para entre cinco y seis de las preguntas se clasificaron como de calidad metodológica media; y los estudios que recibieron un «sí» para cuatro o menos se clasificaron como de baja calidad metodológica. Por otro lado, los estudios cualitativos que recibieron un «sí» a ocho o más preguntas se clasificaron como de alta calidad metodológica; los que recibieron un «sí» a entre cinco y siete preguntas se clasificaron como de calidad metodológica media; y los estudios que recibieron un «sí» a cuatro o menos preguntas se clasificaron como de baja calidad metodológica. La evaluación fue realizada por dos revisores, C.O.G.L.S. y S.N.R., de forma independiente. Los resultados se compararon y los desacuerdos se resolvieron por consenso.

En cuanto al análisis de los datos extraídos, se realizó una síntesis cualitativa. Se tuvieron en cuenta los atributos metodológicos de los estudios, el resultado medido y los principales resultados obtenidos para crear categorías mayores (conocimientos y satisfacción) y menores (instrumentos para medir los conocimientos y motivos de satisfacción o insatisfacción), según la recurrencia de los hallazgos. Esta categorización resultó del análisis temático^(27)^ de los artículos incluidos en la revisión.

## RESULTADOS

### Selección de Estúdios

De los 1.215 estudios encontrados, se excluyeron 490 por duplicidad, quedando 725 artículos (Figura 1). Tras la lectura de los títulos y resúmenes, se excluyeron 671 estudios por no cumplir los criterios de inclusión, quedando 54 artículos. Tras la lectura completa, se excluyeron 42 estudios porque tampoco cumplían los criterios de inclusión: 11 no trataban sobre DIU, sino de otros MAC; 18 investigaban si la población de estudio sólo conocía la existencia del DIU; 3 no cumplían los criterios de inclusión establecidos para la población de este estudio (uno medía resultados en profesionales de la salud, otro con una muestra de personas no brasileñas y otro con una población de hombres cisgénero); 3 eran duplicados, escritos en portugués e inglés; 4 eran disertaciones (3) o tesis (1), cuyos artículos ya habían sido publicados; 2 no fueron encontrados en su totalidad de forma gratuita; y 1 artículo sólo estudiaba las razones para elegir el DIU como MAC y no abordaba los resultados de interés en esta revisión. Por lo tanto, se incluyeron 12 artículos en esta revisión. Ninguno de los artículos seleccionados fue retractado, y ninguna de las revistas de publicación fue considerada depredadora.

**Figura 1:** Diagrama de flujo para la selección de estudios para la revisión – Brasil, 2024.

De las producciones científicas encontradas, cinco discutieron aspectos relacionados al conocimiento sobre el método (Tablas 1 y 2), mientras que las otras siete estudiaron la satisfacción de las usuarias con el DIU (Tabla 3). Ningún artículo evaluó los dos resultados simultáneamente, ni estudió hombres trans o personas no binarias. Por lo tanto, este estudio sólo analizará los resultados encontrados en poblaciones de mujeres cisgénero y, por lo tanto, utilizará pronombres femeninos para referirse a los hallazgos.

**Tabla 1:** Características de los estudios sobre el conocimiento de las mujeres sobre el Dispositivo Intrauterino – Brasil, 2024.

Primer autor/año y número de citación	Población/muestra	Uso de DIU	Abordaje metodológica y tipo de estudio	Local	JBI**
Camiá/1997(^28^)	100 puérperas	No	Cualitativo con análisis del discurso	Campo Grande, MS	6/10
Espejo/2003(^31^)	472 mujeres entre 30 e 49 años	No	Cuantitativo transversal	Campinas, SP	9/9
Silva-Filho/2016(^30^)	380 mujeres entre 20 e 30 años	s/e*	Cuantitativo transversal	Brasil	4/9
Diniz/2020(^29^)	22 mujeres entre 14 e 24 años	No	Cualitativo con análisis temático	São Luís, MA, Balsas, MA, e Santana do Ipanema, AL	7/10
Borges/2020(^16^)	1.858 mujeres de 18–49 años	s/e*	Cuantitativo transversal	São Paulo, SP, Aracaju, SE e Cuiabá, MT	8/9

Nota: *: Sin especificación;

**: Verificación del riesgo de sesgo utilizando los criterios del JBI; DIU: Dispositivo Intrauterino; MS: Mato Grosso do Sul; SP: São Paulo; MA: Maranhão; AL: Alagoas; SE: Sergipe; MT: Mato Grosso.

**Tabla 2:** Conocimiento del Dispositivo Intrauterino entre las mujeres brasileñas que utilizan o no el método anticonceptivo – Brasil, 2024.

Primer autor/año y no citación	Objetivo	Instrumento	Preguntas	Resultados principales
Camiá/1997(^28^)	Evaluación del nivel de información sobre métodos anticonceptivos entre las puérperas	Cuestionario estructurado con 10 preguntas abiertas y cerradas. No había preguntas específicas sobre el DIU, y podía mencionarse libremente.	¿Has oído cómo evitar el embarazo? (sí/no); ¿Qué has oído para evitar el embarazo? (abierto); ¿Qué sabes? (abierto a hablar de los métodos mencionados).	Correctos: 19,4%* Parcialmente correctos: 41,9%* Incorrecto: 38,7%*.
Espejo/2003(^31^)	Evaluar el conocimiento de los métodos anticonceptivos y su posible asociación con las características socioeconómicas y demográficas.	Cuestionario estructurado con 18 preguntas, 6 de ellas sobre métodos anticonceptivos y sólo 1 sobre el DIU.	Cuando una mujer utiliza un DIU y se queda embarazada, el bebé nace con el DIU pegado. (Cerrado – de acuerdo, en desacuerdo o más o menos).	Me contestaron que, en caso de fallo, el bebé nacería con el DIU pegado (sin porcentaje descrito).
Silva-Filho/ 2016(30)	Crear datos sobre el conocimiento del DIU por parte de las mujeres latinoamericanas.	Encuesta en línea con 8 afirmaciones sobre el DIU con opciones de respuesta «sí» o «no».	1 – He oído historias negativas sobre el DIU en televisión, revistas o periódicos.	Alrededor del 30% declaró haber oído historias negativas sobre el DIU a amigos y familiares.
			2 – He oído historias negativas sobre el DIU en Internet.	Cerca de 30% relataram ter ouvido histórias negativas sobre o DIU de amigos e familiares.
			3 – Amigos y familiares te han contado historias negativas sobre el DIU.	Alrededor del 25% cree que las nulíparas no pueden utilizar el DIU.
			4 – Los profesionales sanitarios te han contado historias negativas sobre el DIU.	Alrededor del 15% de las mujeres está de acuerdo en que el DIU es más eficaz que otros métodos anticonceptivos
			5 – Sólo pueden utilizarlo las mujeres que ya han tenido hijos.	Alrededor del 40% está de acuerdo en que es un método más caro que la mayoría de los demás métodos anticonceptivos.
			6 – Solo pueden llevarlo las mujeres de más edad (40+).	Los demás resultados tenían un valor p > 0,05.
			7 – Es más eficaz que otros métodos anticonceptivos	
			8 – Es más caro que la mayoría de los demás métodos anticonceptivos.	
Diniz/2020(29)	Describir las percepciones y necesidades de las mujeres jóvenes que buscan servicios de salud sexual y reproductiva.	Entrevista semiestructurada que siguió una guía temática**.	Se plantearon cuestiones sobre el virus del Zika y su transmisión sexual, así como sobre anticoncepción, aborto, embarazo y necesidades en salud sexual y reproductiva.	Las mujeres declararon tener miedo a la inserción porque habían oído historias en sus comunidades.
				Asociaron el uso de DIU con el cáncer, el aborto espontáneo, la enfermedad inflamatoria pélvica, la infertilidad y el dolor;
				Además, las mujeres declararon tener miedo a introducirse un objeto extraño en el cuerpo.
Borges/2020(16)	Analizar el nivel de conocimientos sobre el DIU entre las mujeres en edad reproductiva.	Cuestionario estructurado con 6 afirmaciones sobre el DIU con opciones de respuesta «de acuerdo», «en desacuerdo», «no sé»	1 – El hombre siente el DIU durante el coito.	1 – El 53,7% no pudo responder si el hombre siente el DIU durante el coito y el 4,9% estuvo de acuerdo.
			2 – El DIU se coloca mediante cirugía.	2 – El 24,3% creía que el DIU se coloca mediante cirugía y el 26,1% no supo responder.
			3 – Los DIU son abortivos.	3 – El 13,7% estuvo de acuerdo en que el DIU es abortivo y el 32,2% no supo responder.
			4 – Una vez retirado el DIU, la mujer tiene dificultades para quedarse embarazada.	4 – El 13,3% estuvo de acuerdo en que sería difícil que una mujer se quedara embarazada después de extraer el DIU y el 33,8% no supo responder.
			5 – Los DIU aumentan el riesgo de cáncer de útero.	5 – El 21% creía que el DIU aumenta el riesgo de cáncer de útero y el 41,4% no sabía la respuesta.
			6 – El DIU provoca muchos efectos secundarios desagradables.	6 – El 31,3% estuvo de acuerdo en que el DIU provoca muchos efectos secundarios desagradables y el 39,8% no supo responder.
				Nota: el 7,2% de las mujeres acertaron los seis puntos y el 15,3% los fallaron todos. La mediana del número de respuestas correctas fue de 3.

Nota: DIU: Dispositivo Intrauterino; n^º^: número; MAC: métodos anticonceptivos;

*: Clasificación tomada del artículo original;

**: Las preguntas realizadas según cada tema no estaban descritas en el artículo original.

**Tabla 3:** Satisfacción con el uso del Dispositivo Intrauterino por las mujeres brasileñas usuarias del método – Brasil, 2024.

Primer autor/año y número citación	Población/muestra	Abordaje metodológico y tipo de estudio	Local	Instrumento	Resultado principal	JBI**
Albuquerque/ 2021(^32^)	158 mujeres en post-parto	Cuantitativo cohorte prospectiva	Fortaleza, CE	Cuestionario con escalas Likert	6sem: 92,4% satisfechas 6m: 86,9% satisfechas	6/9
Scavuzzi/2016(^33^)	157 sin embarazos y mujeres con parto anterior	Cuantitativo transversal	Recife, PE	Cuestionario estructurado con escala 0–10 de satisfacción	Nulíparas: 77,8% completamente satisfechas C/ parto anterior: 76,7% completamente satisfechas	6/9
Laporte/2022(^38^)	517 mujeres usuarias de DIU hormonal	Cuantitativo transversal	Campinas, SP	Cuestionario cerrado	91% das mujeres satisfechas con el DIU 97% recomendarían para otras mujeres	4/9
Oliveira/2023(^34^)	100 mujeres entre 15 y 24 años	Cuantitativo cohorte prospectiva	Belo Horizonte, MG	Cuestionario estructurado	12m: 93,9% satisfechas 24m: 100% satisfechas 36m: 96,8% satisfechas	5/9
Carvalho/2017(^35^)	251 mujeres usuarias de DIU hormonal entre 18 e 45 anos	Cuantitativo Cohorte Prospectiva	Campinas, SP	Cuestionario estructurado	12m: 93,1% muy satisfechas o satisfechas*	5/9
Giovanelli/2021(^36^)	294 adolescentes con <19años	Cuantitativo Cohorte Prospectiva	São Bernardo do Campo, SP	Cuestionario estructurado	12m: 85,7% satisfechas*	5/9
Borges/2017(^37^)	668 mujeres de 18–49 anos	Cuantitativo transversal	São Paulo, SP	Cuestionario estructurado	Usuarias de DIU: 94,7% satisfechas	9/9

Nota: m: meses; sem: semanas; MAC: métodos anticonceptivos;

*: Aunque el estudio fue definido por los autores como una cohorte prospectiva, el resultado de interés en esta revisión se midió en un solo punto temporal.

**: Verificación del riesgo de sesgo mediante los criterios del JBI.

## CONOCIMIENTOS

Los cinco estudios sobre el conocimiento del DIU por parte de las mujeres estaban fechados entre 1997 y 2020, y sólo dos se publicaron en los últimos cinco años (Tabla 1). En cuanto al enfoque metodológico, tres eran estudios cuantitativos transversales y dos cualitativos, uno mediante análisis temático y el otro mediante análisis del discurso. Los estudios investigaron los conocimientos en no usuarias del método (n = 3) y en mujeres que utilizaban o no el DIU (n = 2).

El tamaño de la muestra de estos estudios varió entre 22 y 1.858 mujeres. Los estudios evaluaron a mujeres de entre 14 y 49 años, pero cada uno con un rango de edad diferente. Sólo un estudio consideró una muestra proveniente de Brasil, independientemente de la región. Los demás fueron estudios con muestras locales o regionales, como interestatales (n = 2) o dentro de un único municipio (n = 2). De los estudios realizados en municipios, uno era del estado de São Paulo y otro de Mato Grosso do Sul (Tabla 1).

En los estudios que evaluaron el conocimiento sobre el DIU, fue evidente la desinformación sobre el método, como la creencia de que el DIU es abortivo^(16,28,29)^; causa cáncer^(28,29)^, enfermedad inflamatoria pélvica^(29,30)^ y embarazo ectópico^(30)^; dificulta el embarazo después de la extracción o causa infertilidad^(16)^; sólo es indicado para mujeres que ya tuvieron hijo(s)^(30)^; y que, en caso de falla, el bebé nacerá con el DIU pegado^(31)^ (Tabla 2).

Las mujeres declararon haber oído historias negativas sobre el DIU a amigas, familiares e incluso profesionales de salud^(29,30)^. Además, el miedo a la inserción, a introducir un objeto extraño en el cuerpo y al dolor en el momento de la inserción o durante el uso, fueron señalados por las mujeres como razones de su temor a adoptar el método^(29)^. Se constataron lagunas de conocimiento sobre el lugar de inserción del DIU^(28)^ y sobre lo que es el método, ya que las mujeres creían que era un anillo o un disco, por ejemplo^(28)^.

En un estudio cualitativo^(28)^, los autores crearon categorías para clasificar las respuestas en correctas, parcialmente correctas o incorrectas. En este estudio, se consideró que las mujeres eran correctas cuando decían que el DIU es un dispositivo que el médico coloca en el útero y que debe revisarse periódicamente. Se consideró parcialmente correcto cuando informaron de que se insertaba en el interior de la mujer, en el cuello uterino o en la vagina, que era similar a un sedal con dos extremos y que era abortivo. Por último, se consideró incorrecto cuando consideraron que el DIU era similar a una piel, que impedía el embarazo y las enfermedades; que era un anillo cubierto de pomada que se insertaba en el útero; y que era un disco colocado en la entrada del útero que funcionaba como una tapa. Considerando estas categorías, el 19,4% tenía conocimientos correctos sobre el DIU, el 41,9% conocimientos parcialmente correctos y el 38,7% conocimientos incorrectos (Tabla 2).

En un estudio sobre los conocimientos de las usuarias de los centros de salud acerca del DIU^(16)^ se plantearon a las mujeres seis preguntas a las que podían responder con «estoy de acuerdo», «no estoy de acuerdo» o «no lo sé»: 1) el DIU es abortivo; 2) una vez retirado el DIU, es difícil que la mujer se quede embarazada; 3) el DIU se coloca mediante cirugía; 4) el hombre siente el DIU durante las relaciones sexuales; 5) el DIU aumenta el riesgo de cáncer de útero; 6) el DIU provoca muchos efectos secundarios desagradables. De las 1.858 mujeres de la muestra, sólo el 7,2% acertó los seis ítems y el 15,3% se equivocó en todos, con una mediana de tres (Tabla 2).

### Satisfacción

Siete estudios investigaron la satisfacción de los usuarios con el método y fueron publicados entre 2016 y 2022, cuatro de ellos en los últimos cinco años (Tabla 3). En cuanto al enfoque metodológico, todos los estudios fueron cuantitativos, con predominio de estudios prospectivos (n = 4), seguidos de estudios transversales (n = 3). El tamaño muestral de los estudios varió entre 100 y 668 mujeres. Los estudios analizaron a mujeres de entre 15 y 49 años, cada uno con diferentes grupos de edad. Todos utilizaron muestras locales a nivel municipal. De ellos, cuatro estudios se realizaron en el estado de São Paulo, uno en Minas Gerais, uno en Ceará y uno en Pernambuco.

En los estudios con población brasileña usuaria del DIU se encontraron altos porcentajes de satisfacción con el uso del DIU, a saber: 92,4% y 86,9% estaban satisfechas 6 semanas y 6 meses después de la inserción, respectivamente^(32)^; 77,8% de las nulíparas y 76,7% de las parturientas estaban completamente satisfechas^(33)^; 93,9%, 100% y 96,8% satisfechas a los 12, 24 y 36 meses de uso, respectivamente^(34)^; 93,1% satisfechas a los 12 meses de la inserción^(35)^; 85,7% satisfechas a los 12 meses después del parto^(36)^; 94,7%^(37)^ y 91%^(38)^ satisfechas, independientemente del momento de uso.

 De los siete artículos que estudiaron la satisfacción de las usuarias, cuatro investigaron los factores relacionados con la insatisfacción con el uso del DIU; dos estudios analizaron los factores relacionados con la satisfacción con el método; y un estudio analizó las razones de la satisfacción y la insatisfacción. Los resultados se resumen en la Tabla 4.

**Tabla 4:** Factores relacionados a la (in)satisfacción con el uso del Dispositivo Intrauterino entre mujeres brasileñas – Brasil, 2024.

Insatisfacción	Número de citación
Aumento o prolongación del sangrado menstrual	(32,35,36)
Dismenorrea	(32,36)
Hemorragia intermenstrual	(35)
Otros efectos secundarios (como acné, aumento de peso y migrañas)	(35)
**Satisfacción**	**Número de citación**
Amenorrea o disminución del flujo menstrual	(35,38)
Confianza en el método	(33)
Dismenorrea disminuida	(38)
Consulta con un profesional de salud antes de utilizar el método	(37)

## DISCUSIÓN

Los hallazgos de este estudio señalan la estigmatización del DIU y las lagunas de conocimiento sobre el mismo, factores que pueden incidir en el deseo de las mujeres de utilizar el método en el país. Se destaca la falta de preguntas estandarizadas o de aspectos a ser medidos para evaluar el conocimiento sobre el método, lo que en cierta medida compromete la comparación de los resultados de los estudios existentes. Sin embargo, esos hallazgos en estudios brasileños corroboran los de una revisión sistemática de estudios de otros países, en los cuales las mujeres también asociaron el DIU a infertilidad, embarazo ectópico y cáncer, así como a la creencia de que el método no podría ser utilizado por mujeres nulíparas, por ejemplo, especialmente en países de renta baja y media de Asia y África^(39)^.

En el presente estudio, las razones para no utilizar el método fueron el miedo a introducir un objeto extraño en el cuerpo y el miedo al procedimiento de inserción. Estos resultados corroboran un estudio realizado en Corea que investigó las percepciones de las mujeres sobre el DIU^(40)^. Además, las mujeres brasileñas manifestaron miedo al dolor durante la inserción, lo que favoreció la no utilización. Sin embargo, un estudio realizado en São Paulo con mujeres que se habían sometido a la inserción del DIU reveló que menos del 19% calificaron el dolor de intenso, y la mayoría calificó el dolor de moderado (44%) y leve (37,3%)^(41)^. Por otra parte, el dolor es una experiencia individual y puede estar presente durante la inserción del DIU, por lo que los profesionales deben informar a las mujeres sobre las estrategias para mitigarlo, incluida la oferta de analgesia^(42)^.

La falta de estos conocimientos y prácticas también puede contribuir a un menor uso del método. Además, los hallazgos también refuerzan el hecho de que la falta de conocimiento y los conceptos erróneos sobre el método y su inserción pueden estimular temores exagerados, causando daños a las propias mujeres, ya que no utilizan el método debido a estas cuestiones. Así, los resultados encontrados en relación al conocimiento inadecuado sobre el DIU entre las mujeres brasileñas indican que el acceso a los servicios de planificación reproductiva, especialmente el acceso a informaciones adecuadas sobre este método, parece estar por debajo de lo recomendado^(43)^.

A pesar del conocimiento bajo y incorrecto sobre el método, cabe destacar que las mujeres están interesadas en recibir más información. El estudio *Thinking About Needs in Contraception*, una encuesta cuantitativa *online* sobre las opiniones de profesionales sanitarios y mujeres sobre aspectos del asesoramiento anticonceptivo y el uso de anticonceptivos, encontró que el 66% de las mujeres estaban interesadas en recibir más información sobre todos los métodos y que el 69% considerarían el uso de LARC si recibieran una información más completa^(44)^. Por lo tanto, se reitera que si las mujeres recibieran información adecuada, se podrían aliviar las preocupaciones y los temores señalados en los estudios.

Incluso con la escasa evaluación de la fuente de desinformación sobre el DIU en los estudios, no es irreal suponer que las mujeres pueden haber buscado en Internet respuestas a sus dudas. En este contexto, es necesario considerar la falta de fiabilidad de estos sitios que, incluso cuando están especializados en anticoncepción, difunden información incoherente y engañosa sobre el método, lo que puede contribuir a su no utilización^(45)^. Por otro lado, cuando la información es suministrada por profesionales de la salud capacitados, aumenta el interés por el uso del método. Un estudio realizado en São Paulo encontró que más de 98% de las mujeres a las que se les colocó un DIU por obstetras y enfermeras obstétricas informaron que habían recibido consejos de planificación reproductiva antes de la inserción y que, de ellas, casi todas (98,6%) consideraron que era suficiente para sentirse seguras usando el DIU^(41)^, aunque los estudios de esta revisión hayan mostrado que las mujeres reconocen al profesional médico como el responsable de la inserción.

Las enfermeras formadas tienen el aval para insertar el DIU^(13)^ y, dentro del ámbito de la enfermería, tienen competencia exclusiva para trabajar en planificación familiar y reproductiva^(46)^. Además, un estudio realizado con datos del Sistema de Información Sanitaria de Atención Primaria en 2021 muestra que las enfermeras realizan la mayoría (alrededor del 76%) de las consultas individuales relacionadas con el DIU, mientras que los profesionales médicos realizan aproximadamente el 24% de las consultas de orientación sobre el método^(47)^. Un estudio realizado en Etiopía investigó los factores asociados al uso del DIU después del parto y descubrió que las mujeres que recibían asesoramiento profesional después del parto tenían tres veces más probabilidades de utilizar el DIU que las que no lo hacían^(17)^.

Así pues, es necesario que los profesionales de salud proporcionen a las mujeres información correcta y con base científica. Sin embargo, la literatura dice a respecto de la existencia de un vacío de conocimientos por parte de estos profesionales sobre el DIU. Un estudio realizado en Ciudad del Cabo (Sudáfrica) reveló que alrededor del 14% de los profesionales creían que el DIU puede migrar en el cuerpo, el 11,1% que aumenta la posibilidad de aborto espontáneo, el 18,1% que se asocia a embarazo ectópico y el 22,2% que se asocia a EPI^(15)^.

Tener acceso a conocimientos con base científica sobre el MAC, especialmente por parte de profesionales formados, es fundamental para que las mujeres puedan tomar decisiones sobre su salud sexual y reproductiva, permitiéndoles elegir el método que mejor se adapte a sus necesidades y circunstancias vitales^(18)^. Estas actividades educativas, que incluyen asesoramiento colectivo e individual, son las que se recomiendan en el contexto de la planificación reproductiva^(43)^. Sin embargo, un estudio realizado con una muestra representativa de la población brasileña reveló que, en 2019, el 95,4% de las mujeres no habían participado en grupos de planificación reproductiva^(1)^. El estudio estadounidense CHOICE mostró que un mayor conocimiento y la eliminación de las barreras financieras condujeron a un aumento en el uso, la tasa de continuación y la satisfacción con los anticonceptivos reversibles de acción prolongada^(48)^, lo que refuerza la necesidad de orientación.

Los estudios incluidos en esta revisión también mostraron una alta tasa de satisfacción entre las usuarias de DIU. Sin embargo, faltaban parámetros estandarizados para evaluar el nivel de satisfacción de las usuarias. Se utilizaron diferentes instrumentos, como escalas de Likert, escalas de 0 a 10 y diferentes categorizaciones (satisfecha o insatisfecha; muy satisfecha, satisfecha, insatisfecha o muy insatisfecha; y completamente satisfecha, parcialmente satisfecha o insatisfecha), similar a lo observado para evaluar el conocimiento sobre el método. Hasta cierto punto, esto pone en peligro la comparabilidad de sus resultados entre estudios.

Entre los efectos secundarios, un cambio en el patrón de sangrado fue el determinante más citado de la satisfacción con el uso del DIU. El aumento del sangrado, en términos de frecuencia o intensidad, era un motivo de insatisfacción, mientras que la disminución del sangrado era un factor positivo para la satisfacción. En este sentido, una vez más, el conocimiento previo podría ayudar a las mujeres a identificar y gestionar estos efectos. Sin embargo, estos cambios en el patrón de sangrado no fueron suficientes para reducir la tasa de satisfacción entre las mujeres brasileñas con el uso de DIU, como demuestran las encuestas realizadas en Brasil, la mayoría de las cuales indicaron más de un 80% de satisfacción. Estos índices son incluso superiores a los encontrados en Europa, con 76–78% de muy satisfechas y en Egipto, 77,8% de satisfechas^(22)^.

Se observa, por lo tanto, que el DIU es un método del tipo LARC ampliamente utilizado en países de renta alta, pero poco utilizado en Brasil. El conocimiento de las mujeres brasileñas sobre el método es bajo y rodeado de conceptos erróneos, realidad que también se observa en el mundo en general, lo que podría explicar, en parte, la subutilización del método en Brasil en comparación con el contexto mundial. Por otro lado, cuando se utilizan, el nivel de satisfacción es extremadamente alto, incluso superior al de otros países. Con base en los resultados de este estudio, se puede inferir que existen problemas de acceso, incluyendo el acceso a la información, que puede ser menor que la necesaria, además de la disponibilidad del método en el menú del SUS.

Considerando el interés de las mujeres en recibir información más completa sobre el método^(44)^ y el impacto de esta orientación en la probabilidad de uso^(17)^, los profesionales de salud en Brasil pueden desempeñar un papel fundamental para garantizar el acceso al conocimiento adecuado. En este sentido, la capacitación de los profesionales también parece necesaria, y la participación de enfermeros y enfermeras obstétricas ha sido fundamental para garantizar el derecho y el acceso a este y otros métodos^(41,47)^. Por lo tanto, se espera que, después de consultar a un profesional capacitado, el nivel de conocimiento de las mujeres aumente, lo que también aumentará las posibilidades de uso del DIU. Sin embargo, no se encontró en la literatura ningún estudio que evalúe el conocimiento de las mujeres brasileñas sobre el método antes y después de la consulta con un profesional de salud o de una intervención educativa.

Una limitación del estudio es que las evidencias deben generalizarse con cautela, debido a los diferentes diseños y métodos para medir el conocimiento sobre el método y la satisfacción con él entre las usuarias. Además, la producción nacional sobre el tema se concentra en la ciudad de São Paulo, ya que cinco (41,67%) de los doce estudios incluidos fueron realizados allí. Se trata de una ciudad del estado más desarrollado del país y su población podría presentar diferencias considerables en relación a los demás estados del país, especialmente si se consideran las desigualdades en el acceso a la anticoncepción anteriormente descritas^(1,2)^. Esto se traduce en diferentes elecciones y usos de los MAC disponibles por parte de la población.

Además, ningún estudio ha investigado los resultados en hombres transgénero o personas no binarias. El acceso de esta población es aún más restringido que el de las mujeres cisgénero, que también ha sido limitado. Un estudio señala que los grupos marginados tienen mayores dificultades para acceder a los servicios de salud sexual y reproductiva^(49)^. Por lo tanto, no es irreal inferir que este grupo tiene menos acceso a la información y, por lo tanto, un conocimiento aún menor que el encontrado en los estudios incluidos en esta revisión, lo que puede resultar en una menor probabilidad de uso por parte de los hombres trans y las personas no binarias. Por lo tanto, se necesitan estudios que evalúen el conocimiento en esta población, así como estrategias que sean inclusivas para garantizar el uso.

Además, una revisión sistemática depende de la calidad de los estudios incluidos. Al analizar la calidad metodológica de los estudios, 8 estudios se clasificaron como de calidad media, principalmente debido a la falta de discusión de las tasas de respuesta (los autores deberían discutir claramente la tasa de respuesta y cualquier razón para la falta de respuesta y comparar las personas en el estudio con las que no están en el estudio), así como el uso de diferentes medidas para el conocimiento y diferentes escalas para la satisfacción. Por lo tanto, es necesario que los futuros estudios se refuercen metodológicamente y permitan una mayor comparabilidad entre ellos.

Por otro lado, este estudio supone un avance al identificar lagunas en la literatura sobre la evaluación del conocimiento del DIU por parte de las mujeres. Históricamente, los estudios se han centrado en los mitos sobre el DIU y han medido aspectos relacionados con las creencias de las mujeres, sin estandarizar los resultados. Hace treinta años, el desconocimiento sobre el DIU incluía la apariencia física del método, ya que las mujeres creían que era un anillo o un disco, pero estas concepciones ya no se abordan y pueden ser diferentes en la actualidad. Las investigaciones de hace unos 10 años apuntaban a la creencia de que el DIU sólo es adecuado para mujeres que han tenido hijos, puede provocar embarazos ectópicos o enfermedad inflamatoria pélvica.

La creencia de que el DIU es abortivo y provoca cáncer fue descrita por estudios realizados tanto en 1997 como en 2020, lo que indica que esta concepción se ha mantenido en el tiempo. Por otro lado, la información sobre el conocimiento de los efectos secundarios, la duración, la eficacia y el mecanismo de acción no fue cubierta, independientemente del marco temporal. Estudios actualizados sobre las concepciones, conocimiento y satisfacción de las personas con útero que usan el DIU son necesarios en vista de: estas lagunas identificadas; la evidencia científica más antigua y la persistente falta de información sobre el método; las consecuencias del (no) uso de anticonceptivos, incluso cuando la persona no desea quedar embarazada; y la baja prevalencia de uso del método en Brasil.

Además, al sistematizar las evidencias sobre el conocimiento y la satisfacción de las brasileñas con el método, el estudio puede servir de apoyo para el desarrollo de acciones que mejoren el acceso al método, lo que parece implicar otros obstáculos además del escaso conocimiento. Esta realidad debe ser considerada, especialmente en Brasil, país con territorio continental, desigualdades sociales históricas y gran diversidad cultural y religiosa, factores que pueden estar relacionados con la subutilización del DIU en el país.

## CONCLUSIÓN

Esta revisión ha sintetizado las evidencias científicas sobre el conocimiento de las mujeres brasileñas sobre el DIU y la satisfacción de las usuarias de este método. Los hallazgos indican lagunas en el conocimiento de las mujeres brasileñas sobre el DIU, destacando los conceptos erróneos y los mitos que lo impregnan. El estudio apunta a la estigmatización del método y a la difusión de creencias erróneas, como la asociación con la infertilidad y el cáncer, incluso por parte de profesionales. Estos resultados ponen de relieve la necesidad de reforzar y mejorar la orientación por parte de los profesionales sanitarios, lo que incluye su formación y el objetivo de mejorar los conocimientos promoviendo el acceso a información precisa y con base científica. Esta orientación debe llevarse a cabo en el contexto de las acciones de planificación reproductiva recomendadas, que también deben reforzarse.

A pesar de este vacío de conocimiento, las mujeres que utilizaron el método declararon altos índices de satisfacción, a pesar de las preocupaciones y la desinformación previas. Aunque hay variaciones en los instrumentos utilizados para evaluar la satisfacción, los resultados sugieren que, una vez superadas las barreras iniciales, el DIU es ampliamente aceptado por las mujeres brasileñas. En última instancia, garantizar el acceso a información precisa es fundamental para asegurar que las mujeres puedan tomar decisiones libres e informadas sobre su salud reproductiva, promoviendo una mayor satisfacción y continuidad en el uso.

## Data Availability

Todo el conjunto de datos que apoya los resultados de este estudio fue publicado en el próprio artículo.
